# Essential role of ROS – 8-Nitro-cGMP signaling in long-term memory of motor learning and cerebellar synaptic plasticity

**DOI:** 10.1016/j.redox.2024.103053

**Published:** 2024-02-01

**Authors:** Sho Kakizawa, Tomoko Arasaki, Ayano Yoshida, Ayami Sato, Yuka Takino, Akihito Ishigami, Takaaki Akaike, Shuichi Yanai, Shogo Endo

**Affiliations:** a Department of Biological Chemistry, Graduate School and Faculty of Pharmaceutical Sciences, Kyoto University, Kyoto, 606-8501, Japan; b Aging Neuroscience Research Team, Tokyo Metropolitan Institute for Geriatrics and Gerontology, Tokyo, 173-0015, Japan; c Molecular Regulation of Aging, Tokyo Metropolitan Institute for Geriatrics and Gerontology, Tokyo, 173-0015, Japan; d Department of Environmental Medicine and Molecular Toxicology, Tohoku University Graduate School of Medicine, Sendai, 980-8575, Japan

**Keywords:** Reactive oxygen species, Motor learning, Synaptic plasticity, Cerebellum, Nitric oxide, Antioxidant

## Abstract

Although reactive oxygen species (ROS) are known to have harmful effects in organisms, recent studies have demonstrated expression of ROS synthases at various parts of the organisms and the controlled ROS generation, suggesting possible involvement of ROS signaling in physiological events of individuals. However, physiological roles of ROS in the CNS, including functional roles in higher brain functions or neuronal activity-dependent ROS production, remain to be elucidated. Here, we demonstrated involvement of ROS – 8-NO_2_-cGMP signaling in motor learning and synaptic plasticity in the cerebellum. In the presence of inhibitors of ROS signal or ROS synthases, cerebellar motor learning was impaired, and the stimulus inducing long-term depression (LTD), cellular basis for the motor learning, failed to induce LTD but induced long-term potentiation (LTP)-like change at cerebellar synapses. Furthermore, ROS was produced by LTD-inducing stimulus in enzyme-dependent manner, and excess administration of the antioxidant vitamin E impaired cerebellar motor learning, suggesting beneficial roles of endogenous ROS in the learning. As a downstream signal, involvement of 8-NO_2_-cGMP in motor learning and cerebellar LTD were also revealed. These findings indicate that ROS – 8-NO_2_-cGMP signal is activated by neuronal activity and is essential for cerebellum-dependent motor learning and synaptic plasticity, demonstrating involvement of the signal in physiological function of brain systems.

## Highlights

• **ROS plays an essential role in motor memory and long-term depression in the cerebellum.**
• Neuronal activity triggers ROS production in the cerebellar Purkinje cells.
• The ROS - 8-NO_2_-cGMP pathway regulates motor memory and long-term depression in the cerebellum.
• The ROS - 8-NO_2_-cGMP pathway inhibits cerebellar long-term potentiation.
• Excessive vitamin E administration impairs motor memory and motor coordination.

## Graphical abstract

**Figure:**
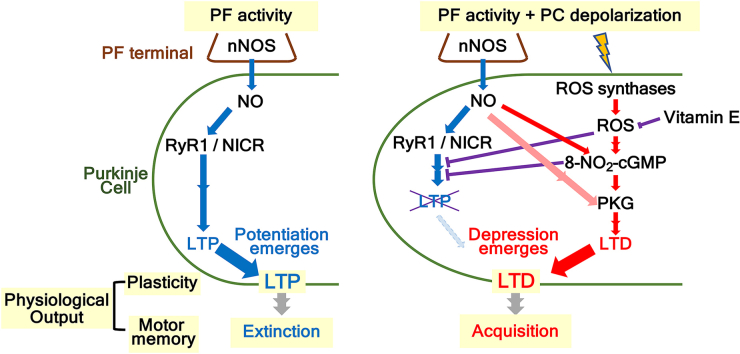
Essential Role of ROS in Acquisition and Directioning of Motor Memory

## Abbreviations:

8-NO_2_-cGMP: 8-nitroguanosine-3′,5′-cyclic monophosphate
8-NO_2_-cGMPS: 8-nitrogyanosine-Rp-3′,5′-cyclic monophosphorothioate
BS: burst stimulation
CF: climbing fiber
CJS: conjunctive stimulation
CNS: central nervous system
DUOX: dual oxidase
EPSC: excitatory postsynaptic current
HRP: horse radish peroxidase
LTD: long-term depression
LTP: long-term potentiation
NICR: NO-induced Ca^2+^ release
NO: nitric oxide
NOX: nicotinamide adenine dinucleotide phosphate oxidase
OKR: optokinetic response
PC: Purkinje cell
PDE: phosphodiesterase
PEG: polyethylene glycol
PF: parallel fiber
PKG: cGMP-dependent protein kinase
RyR1: type 1 ryanodine receptor
SOD: superoxide dismutase
VC: vitamin C (ascorbic acid)
VE: vitamin E (α-tocopherol)

## 1 Introduction

In 1956, Denham Harman hypothesized that the reactive oxygen species (ROS)-induced oxidative stress leads to aging and diseases [1]. However, recent studies demonstrate that ROS is actively generated by enzymes, such as nicotinamide adenine dinucleotide phosphate oxidase (NOX) and dual oxidase (DUOX), in a controlled and signal-dependent manner [2,3], suggesting possible involvement of ROS in various biological events. Actually, beneficial roles of ROS in some tissues and cells, including those for immune system for example, are suggested by recent studies, and the insufficient ROS due to genetic mutation lead to diseases (For recent review, see Ref. [4]). However, although expression of NOX and DUOX are demonstrated in the central nervous system (CNS), the physiological roles of ROS in the CNS have not been clarified well [5,6]. Especially, ROS production by physiological patterns of neuronal activity and the involvement of endogenous ROS in learning and memory have not been demonstrated, and the physiological roles of neuronal ROS in controlling motor functions remain unknown.

Another line of studies also suggests possible involvement of ROS signals in physiological functions of the CNS. ROS scavengers such as ascorbic acid (vitamin C [VC]) and vitamin E (VE) are believed to be beneficial for scavenging ROS in our systems; they are commonly and widely used for achievement purposes in athletes and for health promotion purposes in the general public [7]. However, recent reports have indicated that higher doses of antioxidants can cause harmful effects such as nulling physical exercise and endurance training in humans [[8], [9], [10]]. Recent reviews have also cautioned on the use of VC or VE because of their inconsistent effects, and VC and VE have been shown to impair athletes' training effects [11]. These contradictory roles of VC/VE may be attributed to the complex nature of the motor system examined. We hypothesized that ROS is involved in long-term memory because they can induce long-term effects on the functions of targets through stable chemical modifications. However, despite intense investigation of the molecular mechanisms underlying neuronal plasticity and memory, few studies have examined the role of ROS in motor coordination and memory, which play important roles not only in athletes’ performance but also in skilled learning in general [12].

The cerebellum is the center for motor coordination and motor learning [[12], [13], [14]]. While the cerebellum receives inputs from virtually all the sensory modalities, cerebellar Purkinje cells (PCs) are the sole origin of output from the cerebellar cortex. The outputs from PCs are regulated by two excitatory inputs from parallel fiber (PF) and climbing fiber (CF) [15,16]. Two types of plasticity are observed in PF-to-PC synapse, long-term depression (LTD) and long-term potentiation (LTP), and these plasticities are regarded as the cellular basis for motor coordination and motor learning [17], i.e., LTD for acquisition and LTP for the extinction, respectively. The involvement of some signaling pathways have been identified in cerebellar LTD and LTP [12], however, the molecular mechanisms underlying those plasticities remain to be elucidated. Considering that ROS has the potential to induce prolonged impacts on target molecules [[2], [3], [4]] and that the ROS synthases including NOX and DUOX are expressed in the cerebellum [[18], [19], [20]], it is conceivable that ROS signaling may play a role in cerebellum-dependent motor learning and cerebellar LTD, a long-lasting plasticity. In this study, we tested the hypothesis that ROS controls motor functions and neuronal plasticity in the cerebellum by utilizing simple motor memory (i.e., optokinetic response [OKR] adaptation and rotor rod test) and neuronal plasticity (LTD and LTP) in the cerebellum. Furthermore, we investigated the downstream molecular components underlying the physiological role of ROS.

## 2 Materials and methods

### 2.1 Ethics statement

All animal experiments were approved by the Animal Experiment Committee of the Tokyo Metropolitan Institute for Geriatrics and Gerontology (Animal Protocol Approval nos. 17012, 20018, 23001, 23012 for SE) and Kyoto University (Animal Protocol Approval no. 17-7-6 and 23-7-1 for SK) and were carried out according to the guidelines in *The Guide for the Care and Use of Laboratory Animal* [21]. In this study, efforts were made to describe and report the details of animal experiments according to the Animals Research: Reporting *In Vivo* Experiments (ARRIVE) guidelines [22]. The health status of the mice was monitored daily by animal technicians and appropriate actions were taken if there were any symptoms of ill health. Mice were euthanized humanely if they showed rapid and continuous weight loss or signs of emaciation. At the end of the experiments, the mice were anesthetized using isoflurane in a bell jar. Deep anesthesia was confirmed in the mice by assuring the absence of a pedal reflex (firm toe pinch). When breathing stopped or deep anesthesia was confirmed, the mouse was immediately euthanized by cervical dislocation; the experimenter was formally approved for this procedure [23]. Efforts were made to minimize the use of mice for experiments by considering the 3Rs (ref [24]; see also section below). The numbers of mice used in each experiment are listed in Table S1. Some mice were also used for immunohistochemistry and biochemical analyses in order to reduce the total number of mice used.

### 2.2 Experimental model, subject details and statistics

To estimate the required sample size needed for detecting a statistically meaningful effect for behavioral experiments, we performed an *a priori* power analysis using G∗Power software [25]. For this determination, we assumed that one-way analysis of variance (ANOVA) would be used and a signiﬁcance level of *P* < 0.05 in order to detect a meaningful effect with 80 % actual power (effect size = 0.5). This analysis produced a needed sample size of 74 mice. From the perspective of the 3Rs [24] and on the basis of our experiences with mouse deaths from our previous experiments, we decided to use 40 mice. Additional 22 mice were used for flocculus injection experiments. For the experiments for vitamin C (VC) and Vitamin E (VE) assay, 10 mice/group was used.

The sample sizes of electrophysiological experiments and imaging study were determined with reference to those in our previous studies [[26], [27], [28]], and decided to use at least 3 mice in each experimental group. That made us obtain the electrophysiological data (recordings) from 4 to 10 cells in each group.

For biochemical experiments using behaviorally-trained (OKR) mice, the same power assumption for behavioral analyses yielded a needed sample size of 28 mice each for the OKR training group and the non-training sham group. We reduced the number of mice to 20 mice based on outcomes of our previous similar experiments [29]. Moreover, to maximize data we obtained per mouse, we collected tissue samples from both the flocculi and paraflocculi of the same animal for the biochemical analysis. This allowed us to reduce the use of mice needed and did not compromise answering our experimental questions.

For the induction of an immediate early gene product c-Fos by OKR training, 14 mice were used based on the power analysis above. Ten mice were used to elucidate the distribution of 8-NO_2_-cGMP and S-guanylated proteins.

Statistical differences between groups were evaluated using one-way or two-way ANOVA or two-tailed Student's *t*-test (SPSS, IBM, Japan). When the main effect was statistically significant with an ANOVA, we performed appropriate *post-hoc* tests. Statistical significance was set at *p* < 0.05.

### 2.3 Subjects

Experimentally nave C57BL/6J mice (CLEA Japan Inc., Tokyo, Japan) were used (Table S1). The mice were housed in a specific pathogen-free vivarium in groups of five per cage, which had solid walls made of polysulfone (Techniplast SpA, Buguggiate, Italy); the cage floor was covered with paper-chip bedding material. Since environmental enrichment objects can increase within-cage aggression [30], enrichment materials were not used. The vivarium was maintained at 23 ± 1 °C and 50 ± 5 % humidity under a 12-h light-dark cycle (lights on at 7 a.m.). Periodic examination of microbial status ensured that the vivarium maintained specific pathogen-free quality throughout the study. The mice had free access to food and water throughout the experiment, unless otherwise mentioned (see the next paragraph).

For Regular diet conditions, mice were fed bottled water containing 10 μM EDTA (ethylenediaminetetraacetic acid) and CRF-1 (containing 20 mg VE [tocopherols and tocotrienols] and 16.6 mg VC per 100 g diet). For the Excess VC/VE diet condition, VE content was increased to 54 mg/100 g of CRF-1, and bottled water containing 1.5 g/L of VC and 10 μM EDTA was supplied to mice [31]. Details of ingredients contained in Regular diet (CRF-1) and the Excess VC/VE diet used in current study are shown in Tables S2 and S3, respectively. The supplemented bottled water was replaced with fresh water twice a week. All experiments were performed in a manner blinded to the experimenter.

### 2.4 Reagents

The compound 8-nitroguanosine-3′,5′-cyclic monophosphate (8-NO_2_-cGMP), antibodies against 8-NO_2_-cGMP and S-guanylated proteins were described previously [32]. We synthesized and characterized 8-nitroguanosine-Rp-3′,5′-cyclic monophosphorothioate (8-NO_2_-cGMPS) as described previously [33]. Antibodies against G-substrate and phosphorylated G-substrate were previously described [34,35]. Anti-calbindin antibody was obtained from Swant AG (Burgdorf, Switzerland). Anti PKGIα C-terminal antibody (ab37709) was obtained from Abcam (Cambridge, UK). Anti c-Fos antibody (Ab-2) was obtained from Merck-Millipore (Japan).

Alexa Fluor 647-labeled rabbit anti-NeuN antibody for cerebellar granule cells (ab190565), Alexa Fluor 488-labeled rabbit anti-GFAP antibody for glia (ab302977) were obtained from abcam (Cambridge, UK). Alexa Fluor 594-labeled rabbit anti-calbindin antibody (CST88831) was obtained from Cell Signaling Technology (Danvers MA, USA). Polyethylene glycol-labeled superoxide dismutase (PEG-SOD) and polyethylene glycol-labeled catalase (PEG-catalase) were obtained from Sigma-Aldrich (St. Louis, MO, USA). SOD, catalase and horse radish peroxidase (HRP) for electrophysiology were obtained from Wako Pure Chemicals, Japan.

### 2.5 Immunohistochemistry

Mice were deeply anesthetized with isoflurane and transcardially perfused with phosphate-buffered saline (PBS) followed by 4 % paraformaldehyde in PBS. The brains were removed from the skull and cryoprotected in 20 % sucrose. Sections (20 μm) were immunostained with antibodies against 8-NO_2_-cGMP, S-guanylation, and/or PC markers (G-substrate or calbindin). In sections treated with antibodies raised in mice, M.O.M. Detection Kits (Vector Laboratories, Burlingame, CA, USA) were used to suppress non-specific background immunoreactions. Immunoreactions were visualized using a species-appropriate secondary antibody, an ABC kit, and diaminobenzidine (DAB) as a substrate (Vector Laboratories) or Alexa Fluor 488- or 546-labeled secondary antibodies (Life Technologies, Thermo Fisher Scientific, Waltham, MA, USA). To identify all cells in a section, regardless of the antigen of interest, we stained the slices with 4′,6-diamidino-2-phenylindole (DAPI; Thermo Fisher Scientific), a nuclei-specific stain. When using DAB to visualize immunostaining, the sections were counterstained with eosin. The localization of 8-NO_2_-cGMP was further examined in the mice brain slices (30 μm; Fig. S2) using antibodies against 8-NO_2_-cGMP with Alexa Fluor 405-labeled anti-mouse IgG, Alexa Fluor 594-labeled rabbit anti-calbindin antibody (CST88831) for PCs, Alexa Fluor 647-labeled rabbit anti-NeuN antibody (ab190565) for cerebellar granule cells (GCs), Alexa Fluor 488-labeled rabbit anti-GFAP antibody (ab302977) for glial cells. Fluorescent images were acquired using a confocal fluorescence microscope (FV10i) and Olympus Fluoview software (Olympus, Tokyo, Japan).

For the analyses of c-Fos, an immediate early gene product, the fixed and cryoprotected cerebellum was coronally sectioned at a thickness of 30 μm using a cryostat (Leica Biosystems, Wetzlar, Germany). Cerebellar sections underwent a series of steps, including washing and blocking of endogenous peroxidase with 0.3 % H_2_O_2_ and 0.3 % normal goat serum (NGS; Vector Laboratories, Burlingame, CA, United States) in PBS for 10 min. This was followed by blocking of non-speciﬁc binding and permeabilization with 1.5 % NGS and 0.3 % Triton X-100 in PBS for 2 h. Blocked sections were then incubated overnight at room temperature with rabbit anti-*c*-Fos antibody in PBS containing 0.5 % NGS and 0.3 % Triton X-100. Immunoreactions were visualized using an ABC kit (Vector Laboratories, Newark, CA, USA) and 3,3′-diaminobenzidine as a substrate. Subsequently, the stained sections were briefly counter-stained with eosin. Nissl staining was carried out on the serial sections to identify the location of PCs (Fig. S2I).

For the quantitation of c-Fos-positive cells in the cerebellar slices, digital images were captured by using the virtual whole-slide imaging system (NanoZoomer 2.0-RS; Hamamatsu Photonics, Hamamatsu, Japan). The obtained images were then analyzed to determine the numbers of c-Fos-positive cells. Subsequently, adjacent sections underwent Nissl staining to identify the PC layer (Fig. S2I). The c-Fos-positive PCs were counted in the PC layer (see Fig. S2I) for both flocculus and paraflocculus. The resulting cell number was then converted into the number of cells number per mm length along the single layer of PCs.

### 2.6 Eye movement recordings

Surgery for OKR was done while the mice were under general anesthesia, as is standard practice. Under isoflurane inhalation anesthesia and under aseptic conditions, a platform for head fixation was surgically attached on the cranial bone using a long bolt and synthetic resin [36,37]. After a recovery period of more than 2 days, the operated mice were handled by the experimenter to habituate the mice to the experimental conditions.

For eye movement recordings, individual mice were mounted on the platform with head fixed using the attached fixation bolt; the mouse's body was loosely restrained in a plastic cylinder. An infrared camera was used to record eye movements once the head was fixed [36,37]. OKR was tested by projecting a sinusoidal oscillation of the checked-pattern screen (screen diameter, 60 cm; screen height, 60 cm from the eye of the mouse; check size, 4°). More than 10 cycles of evoked eye movements free from eye blinks and saccades were averaged and used to calculate the mean amplitude using modified Fourier analysis [38]. The gain of eye movement (i.e., OKR adaptation) was defined as the ratio of the peak-to-peak amplitude of eye movements to that of screen oscillation.

The 4-h OKR training consisted of 4 sets of 200 cycles of sustained sinusoidal screen oscillation at 15° and 0.17 Hz (maximum screen velocity, 7.9°/s; ref. [37]) with a rest period (1 h long) in the dark between each set. Short-term OKR adaptation was measured at the end of the 4-h training. The mice were kept in the dark in their cages after 4-h of training. Then, long-term OKR adaptation was assessed 24 h after the end of the 4-h training.

For the analyses of immediate early genes products (c-Fos), the mice were anesthetized with isoflurane immediately after the end of the 4-hr OKR training. Then the mice were transcardially perfused with PBS, followed by 4 % paraformaldehyde in PBS. The brains were removed from the skull and cryoprotected in 20 % sucrose. The brains were processed and stained using antibodies for c-Fos as mentioned in Section 2.5 above.

### 2.7 Rotor rod test

Motor coordination in mice was examined using the rotor-rod task. Mice were individually placed on a rotating rod, according to a previously described protocol [39]. Once the mouse was on the rod (diameter, 32 mm), its axial rotational speed progressively increased from 4 to 40 rotations per minute over a test period of 300 s. Three trials were conducted per day for six consecutive days, with each trial separated by a 15-min intertrial interval.

### 2.8 Pharmacological reagents and experiments

PEG-SOD (2000 U/mL), PEG-catalase (2000 U/mL), or 8-NO_2_-cGMPS (1 mM) were dissolved in saline and infused bilaterally into the flocculi (0.5 μL for each side) using a 1 μL microsyringe (Hamilton Company, Reno, NV, USA) mounted on standard micromanipulators (Narishige Group, Tokyo, Japan). Under isoflurane anesthesia, mice underwent drug infusion, which was completed within 10 min. OKR was measured 1 h after the mice awoke from anesthesia. As a vehicle control, the same amount of saline was infused bilaterally into the flocculi before the 4-h OKR training.

At the end of the OKR experiments, the mice were anesthetized with isoflurane, and then 1 % toluidine blue or fluorescein isothiocyanate was infused bilaterally into the flocculi at the injection site; at histology, we could then assess the accuracy of the drug injections, since the toluidine blue (or fluorescein) would localize approximately at the injection site.

The mice were then transcardially perfused with PBS, followed by 4 % paraformaldehyde in PBS. The brains were removed from the skull and cryoprotected in 20 % sucrose. Cerebellar sections (20 μm) were cut in the sagittal plane and were examined for injection locations under a microscope (Eclipse 80i) using QCapture software (Nikon, Tokyo, Japan) or under a confocal fluorescence microscope (FV10i; Olympus, Tokyo, Japan) using FluoView software. For staining for PEG (for the injection of PEG-SOD and PED-catalase), the sections were immunostained using a rabbit anti-PEG antibody (Abcam, Waltham, MA, USA). Immunoreactions were visualized using an anti-rabbit IgG antibody, an ABC kit (Vector Laboratories), and DAB as a substrate. The stained slices were examined under a microscope (Eclipse 80i) using QCapture software. Mice with injection sites localized outside of the flocculi were excluded from further analyses.

### 2.9 Quantitation of VC and VE

Amounts of VC (ascorbic acid and dehydroascorbic acid) and VE (α-tocopherol) in the cerebellum were measured. The mice were deeply anesthetized with isoflurane, and the cerebellum was carefully dissected out. The cerebellum was weighed and homogenized in 14 vol of 5.4 % metaphosphoric acid containing 1 mM EDTA for VC measurements. The homogenate was centrifuged at 20000×*g* for 10 min at 4 °C. The resulting supernatant was subjected to high-performance liquid chromatography (HPLC) analysis to determine the amount of VC in cerebellum using HPLC and an electrochemical detector [40].

For the VE measurement, the cerebellum was homogenized in 5 vol of PBS (20 mM sodium phosphate containing 0.15 M NaCl, pH 7.4). The amount of VE was measured using HPLC and an electrochemical detector [41].

### 2.10 Biochemical analysis

We assessed cGMP-dependent protein kinase (PKG) S-guanylation and G-substrate phosphorylation in the cerebellum. The cerebellar flocculi and paraflocculi were separately collected from mice that had undergone 4 h of OKR training. Both PKG and G-substrate are concentrated in PCs in cerebellum [[42], [43], [44]]. Cerebellar cytosolic fractions were determined to contain 1.0 pmol/mg of PKG [44] and 27 pmol/mg of G-substrate [42]. In the present study, the flocculi of one mouse yield approximately 0.1 mg of total cytosolic protein, which contains 0.1 pmol of PKG and 2.7 pmol of G-substrate. To overcome the limited amount of PKG and G-substrate needed for further analyses, flocculi from two mice were combined and used as one sample for subsequent analyses.

The mice in the training group were subjected to 4 h of OKR training (for the training method, see *2.6. Eye movement recordings*). The sham control group was not subjected to the 4-h OKR training. Immediately after the 4-h training, the mice were deeply anesthetized with isoflurane, and flocculi and paraflocculi were dissected out and separately collected under the guidance of a stereo microscope. The samples were stored at −80 °C until use.

### 2.11 Analysis of PKG S-guanylation

Flocculi and paraflocculi stored at −80 °C were thawed and homogenized in ice-cold homogenization buffer (HB; 20 mM Tris-HCl, pH 7.5 containing protease inhibitor mix (Complete [Roche Diagnostics, Indianapolis, IN, USA]) and phosphatase inhibitor cocktail (Nacalai Tesque, Kyoto, Japan). Homogenates were centrifuged at 20000×*g* for 20 min at 4 °C, and 8-(2-aminoethylthio)-cGMP (8-AET-cGMP) agarose gel (BIOLOG Life Science Institute, Bremen, Germany) equilibrated in HB was added to the supernatants and gently mixed for 30 min at 4 °C to collect cGMP-binding proteins [45]. Unbound proteins were stored at −80 °C for the analysis of G-substrate phosphorylation.

For the analysis of S-guanylated PKG, first, the 8-AET-cGMP-gel was washed with washing buffer, then with washing buffer containing 1 mM 5′-AMP, and finally with HB. Washing buffer comprised 50 mM Tris-HCl (pH 7.5), 50 mM NaCl, 5 mM sodium phosphate, 1 % Triton X-100, 1X Complete (Roche Diagnostics, Indianapolis, IN, USA), and 1X phosphatase inhibitor cocktail (Nacalai Tesque, Kyoto, Japan). The washed gel was incubated in sodium dodecyl sulfate (SDS)-sample buffer for 3 min at 95 °C. The supernatant was centrifuged at 20000×*g* for 20 min and subjected to SDS-polyacrylamide gel electrophoresis (PAGE). The separated proteins were transferred onto an Immobilon-FL membrane (pore size, 0.45 μm; Merck Millipore, Burlington, MA, USA) using 10 mM CAPS-NaOH (pH 10.5) containing 10 % methanol [46]. The membrane was blocked with 1x Odyssey Blocking Buffer (LI-COR Biosciences, Lincoln, NE, US) at room temperature, and then incubated with a combination of rabbit anti-PKGIα C-terminal antibody (abcam) and mouse anti-S-guanylated Cys antibody [45] overnight at 4 °C. Immunoreactions were visualized with IRDye 800CW-labeled goat anti-mouse IgG and IRDye 680RD-labeled goat anti-rabbit IgG. Fluorescence images were acquired and analyzed using the Odyssey CLx imaging system and Image Studio software (LI-COR Biosciences). The fluorescence signal for S-guanylation was normalized with the PKG1α signal.

### 2.12 Analysis of G-substrate phosphorylation

For the analysis of G-substrate phosphorylation in flocculi, unbound proteins (i.e., proteins not bound to 8-AET-cGMP-gel; see *2.11. Analysis of PKG S-guanylation* above) stored at −80 °C were thawed, and trichloroacetic acid was added to achieve a 10 % (w/v) trichloroacetic acid mixture. The mixture was then centrifuged at 3000×*g* for 10 min. The precipitate was washed with acetone and air dried. The resulting pellet was dissolved in HB supplemented with 0.001 % Brij35, and the solution's pH was adjusted to 7.5. The solution was heated for 5 min at 95 °C. The acid-stable and heat-resistant nature of G-substrate makes it possible to concentrate G-substrate using this extraction procedure [43]. The mixture was centrifuged for 20000×*g* for 20 min. The resulting supernatant was mixed with SDS-denaturing buffer and heated for 3 min at 95 °C. The SDS-treated samples were subjected to SDS-PAGE, and separated proteins were transferred onto PVDF (Polyvinylidene difluoride) membranes (pore size 0.2 μm; ATTO, Tokyo, Japan) using 10 mM CAPS (3-(Cyclohexylamino)-1-propanesulfonic acid)-NaOH (pH 10.5) containing 10 % methanol [46]. The membrane was blocked with 1x Intercept Blocking Buffer (LI-COR Biosciences, Lincoln, NE, USA) at room temperature, and then incubated overnight at 4 °C with a combination of rabbit anti-G-substrate antibody and mouse anti-phosphorylated G-substrate antibody [34,35]. Immunoreactions were visualized with IRDye 800CW-labeled goat anti-mouse IgG and IRDye 680RD-labeled goat anti-rabbit IgG (LI-COR Biosciences, Lincoln, NE, USA) in 0.5x Intercept Blocking Buffer, 0.2 % Tween20, and 0.01 % SDS for 1 h at room temperature. Fluorescence images were acquired and analyzed using the Odyssey CLx imaging system and Image Studio software (LI-COR Biosciences, Lincoln, NE, USA). The fluorescent signal due to phospho-G-substrate was normalized to the signal for total G-substrate.

### 2.13 Phosphodiesterase inhibition by 8-NO_2_-cGMP

Inhibition of phosphodiesterase (PDE) activity was examined through fluorescence polarization using FAM-labeled cAMP (1{2-(6-[Fluoresceneinyl] aminohexylcarbamoyl) adenosine-3,5-cyclic monophosphate}) or FAM-labeled cGMP (1{2-(6-[Fluoresceneinyl] aminohexylcarbamoyl) guanosine-3,5-cyclic monophosphate}) as a substrate in the presence of 8-NO_2_-cGMP or in the presence of a specific PDE inhibitor (1–10000 nM). PDE type, amount of PDE, specific inhibitor, and substrate used are summarized in legend of Fig. S5.

### 2.14 Electrophysiological recordings and LTD induction

Mice were euthanized by cervical dislocation under deep anesthesia with isoflurane. The cerebellum was excised, and parasagittal cerebellar slices (250 μm thick) were prepared from the level of the vermis and maintained *in vitro*. Whole-cell recordings were obtained from visually identified PCs under an upright microscope (BX51WI, Olympus) using a 40 × water-immersion objective at room temperature (23–25 °C) [47,48]. The resistance of patch pipettes was 2.5–4.0 MΩ when filled with the following intracellular solution composed of (in mM): 120 K-gluconate, 5 KCl, 5 NaCl, 1 EGTA, 4 ATP, 0.4 GTP, and 10 HEPES (pH 7.3; adjusted with KOH). For voltage-clamp recording of parallel fiber (PF)-excitatory postsynaptic currents (EPSCs), a pipette solution with the following composition was used (in mM): 130 l-gluconate, 10 KCl, 10 NaCl, 1 EGTA, 4 ATP-Mg, 0.4 GTP-Na, and 10 HEPES (pH 7.3, adjusted with KOH). Standard slice bathing solution was composed of (in mM) 125 NaCl, 2.5 KCl, 2 CaCl_2_, 1 MgSO_4_, 1.25 NaH_2_PO_4_, 26 NaHCO_3_, and 20 glucose. The bathing solution was bubbled continuously with a mixture of 95 % O_2_ and 5 % CO_2_. Bicuculline (10 μM) was always present in saline to block spontaneous inhibitory postsynaptic currents. Square voltage-pulses (0–10 V; duration, 0.1 ms) were applied for focal stimulation (duration, 0.1 ms; amplitude, 0–10 V) through a glass pipette (tip diameter, 5–10 μm) filled with standard bath solution. Membrane potential was held at −90 to −80 mV, after compensation for the liquid junction potential. Ionic currents were recorded using a patch-clamp amplifier (EPC-9; HEKA, Lambrecht/Pfalz, Germany).

Stimulation and online data acquisition were performed using PATCH MASTER software (HEKA Electroniik GmbH, Reutlingen Germany) on a Windows computer. The analog recordings were filtered at 3 kHz and digitized at 20 kHz.

For LTD and LTP experiments, the intensity of the stimulus was adjusted to evoke PF-EPSCs with initial amplitudes between 80 and 150 pA. After obtaining a stable initial recording for at least 10 min, a conjunctive stimulus (CJS) was applied to induce LTD [27]. The CJS protocol comprised 300 single PF stimuli in conjunction with depolarization pulses (−80 to 0 mV, for 50 ms) repeated at 1 Hz. For LTP induction, 60 burst stimulations (BSs; 1 BS, 5 pulses at 50 Hz) were applied to the PF at 1 Hz [49]. Series resistance and membrane resistance were monitored throughout the experiments, and the data were discarded if either of these resistances varied by more than 10 %. The data were also discarded if the slope of the PF-EPSC (exitatory postsynaptic potential) amplitude averaged every minute during the initial recording for 10 min was larger than 2 % or if the amplitude did not stabilize within 20 min after achieving whole-cell recording [49]. PF-EPSC amplitude was normalized to the mean value calculated over the 10 min period prior to CJS or BS.

### 2.15 Ca^2+^ imaging

For intracellular Ca^2+^ imaging in PCs, a calcium-sensitive dye, Oregon Green BAPTA-1 (100 μM; Thermo Fisher Scientific), was dissolved in the intracellular solution and introduced into the cells using a patch pipette, and the concentration of EGTA in the intracellular solution was decreased to 0.5 mM. The patched cells were examined with an upright microscope (BX51WI, Olympus) equipped with a confocal scanning unit and an argon laser (FV300, Olympus) at an excitation wavelength of 488 nm, and 5–9 sequential confocal images obtained at 3–4 μm intervals (z-axis) were acquired every 0.8 s and were projected onto a plane to obtain images of dendrites at 10-s intervals [26,27].

### 2.16 ROS imaging

For intracellular ROS imaging in PCs, Amplex Ultrared (100 μM; Thermo Fisher Scientific) and horseradish peroxidase (HRP; 10 units/mL) were applied to cells using a patch pipette. In the presence of hydrogen peroxide (H_2_O_2_), Amplex Ultrared is catalyzed by peroxidase to produce resorufin, a red fluorescent by-product, which is then is monitored [50]. Because Amplex Ultrared itself does not yield fluorescence, Alexa Fluor 488, a green fluorescent dye, was co-applied with Amplex Ultrared and peroxidase. The cells were examined with an upright microscope (BX51WI, Olympus) equipped with a confocal scanning unit and an argon laser (FV300, Olympus), and 5–9 sequential confocal images obtained at 3–4 μm intervals (z-axis) were acquired every 0.8 s, and were projected onto a plane to obtain images of dendrites at 10-s intervals.

## 3 Results

### 3.1 ROS scavengers disturbed motor memory in the cerebellar flocculi

**Fig. 1:**
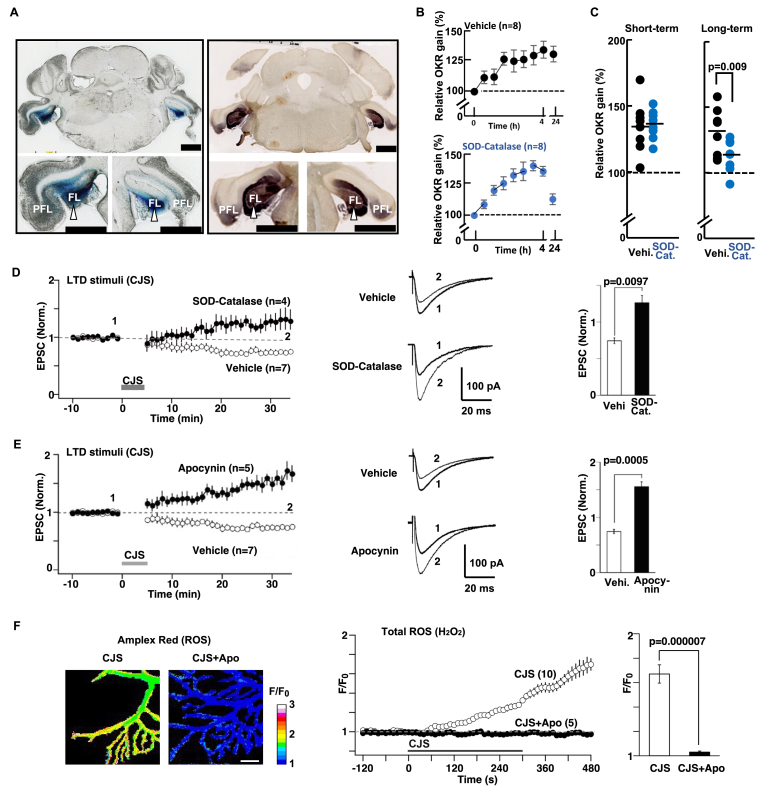
Activity-dependent ROS induction in cerebellar learning and plasticity. **A,** Injection sites of ROS scavengers (SOD-catalase) in mouse flocculi. Left panels: Histological coronal sections (thickness: 20 μm) processed for toluidine blue showing the location of SOD-catalase injection sites into flocculi (FL; arrow head). Paraflocculi (PFL). Right panels: Complementary histological sections immunostained for PEG-SOD-PEG-catalase. Immunoperoxidase reaction due to anti-PEG antibody was visualized with DAB as the substrate. Scale bars, 1 mm. **B,** Time course of OKR training and OKR adaptation after injection of vehicle (Vehi; n = 8) or SOD-catalase (SOD-Cat; n = 8). Repeated measures ANOVA revealed a significant interaction (F [2,19] = 4.1, p = 0.033). The vehicle control is same as the one in Fig. 3A. Thus, statistical analyses were carried out as three groups (vehicle, SOD-catalase, and 8-NO_2_-cGMPS; see also Fig. 3A). **C,** Short- and long-term (4 h and 24 h) relative gain at the end of training. Training protocol is shown in Fig. S1A. **D,** Effect of SOD-catalase on LTD at PF-PC synapses in acute cerebellar slices. SOD-catalase were applied through a patch pipette (SOD, 2000 units/mL; catalase, 2000 units/mL). Left panel: Changes in PF-EPSC recorded from PCs treated with SOD-catalase (n = 4) or vehicle (n = 7) before and after LTD-inducing conjunctive stimulation (LTD stim (CJS)). Middle panel: Representative traces of PF-EPSCs (1) before and (2) 21–30 min after CJS. Right panel: Average amplitude of PF-EPSCs during 21–30 min after CJS. **E,** Effect of apocynin on LTD at PF synapses. Apocynin (250 μM) was applied through patch pipette. Left panel: Changes in PF-EPSC amplitude before and after LTD stim (CJS), recorded from PCs treated with apocynin (n = 5) or vehicle (n = 7). Middle panel: Representative traces of PF-EPSCs (1) before and (2) 21–30 min after CJS. Right panel: Average amplitude of PF-EPSCs during 21–30 min after CJS. **F,** Activity-dependent production of ROS in PCs. Left panels: Pseudocolored images of ROS monitored by a fluorescent ROS probe (Amplex Ultrared) in PCs of cerebellar slices. Fluorescent intensity (F) was normalized by corresponding intensity values before CJS (F_0_). White-to-blue spectrum represents high-to-low levels of fluorescent signal, respectively. Scale bar, 10 μm. Middle panel: Changes in intracellular ROS levels (F/F_0_) in PCs before and after application of CJS in the presence of apocynin (CJS + Apo; n = 5) or vehicle (CJS; n = 10). Right panel: Peak values of CJS-induced elevation of ROS signals in PCs. Data represent mean ± SEM, except for **C** in which percent relative gains of individual animal are plotted, and horizontal bar indicates mean relative gain. (For interpretation of the references to color in this figure legend, the reader is referred to the Web version of this article.)

OKR adaptation is a model of cerebellum-dependent motor learning [36,51], and LTD at the parallel-fiber-to-Purkinje-cell (PF-PC) synapse is associated with this adaptation [12]. To test whether ROS is involved in cerebellar learning, we used the OKR model. We bilaterally injected ROS scavengers, polyethylene glycol (PEG)-conjugated superoxide dismutase (SOD) and catalase (i.e., SOD-catalase), or vehicle (saline [37]) into the cerebellar flocculi of mice (Fig. 1A). The mice were subjected to OKR training and testing for OKR adaptation (Fig. S1A). Repeated measures ANOVA revealed a significant interaction between the two groups' performance at the 4-h and 24-h assessments (Fig. 1B). The simple main effect showed that 24 h after the end of 4-h training, the SOD-catalase group showed significantly less OKR adaptation than the vehicle group (113.0 ± 4.4 and 130.8 ± 6.4 % for SOD-catalase and vehicle, respectively; Fig. 1C and Fig. S1B). At the short-term assessment (4 h), the two groups’ performance was indistinguishable (135.7 ± 2.3 % and 134.3 ± 6.9 % for SOD-catalase and vehicle, respectively; Fig. 1C). Thus, the results indicate that ROS scavengers disrupt cerebellum-dependent long-term memory.

### 3.2 ROS scavengers impaired the induction of LTD

Cerebellar LTD is demonstrated to be the basis for cerebellum-dependent memory [12]. Thus, we tested whether inhibition of ROS signaling also affects cerebellar LTD at the PF-PC synapse in cerebellar slices. Electrical stimulation of PF in conjunction with depolarization of PCs (conjunctive stimulation (CJS); see 2.14 for detail) induces LTD of excitatory postsynaptic currents (EPSCs) in vehicle-treated control slices: the amplitude during the 21–30 min after CJS was depressed to a level of 0.74 ± 0.04-fold of baseline levels (Fig. 1D, open circles). On the other hand, in the presence of SOD-catalase, CJS failed to induce LTD (Fig. 1D, closed circles), and the EPSC amplitude during the 21–30 min after CJS was 1.26 ± 0.13-fold of the baseline level. Thus, in the presence of ROS scavengers, the stimulation paradigm that would have otherwise induced LTD at the PF-PC synapse induces LTP.

The NOX and DUOX are the primary sources of regulated ROS generation [52,53]. Because these ROS synthases are expressed in the cerebellum [[18], [19], [20]], we next examined their possible involvement in cerebellar LTD induction. Application of apocynin (a broad-spectrum antagonist of NOX and DUOX) through patch pipette impaired LTD induction. PF-EPSC amplitudes during the 21–30 min after CJS were 1.56 ± 0.11-fold of baseline levels (Fig. 1E). In contrast, a clear decrease in EPSCs (i.e., LTD) was observed in the control group (0.74 ± 0.04-fold). Taken together, these results indicate that ROS synthases and ROS in PC are essential for the cerebellar LTD.

### 3.3 Production of ROS by neuronal activity inducing cerebellar LTD

Impairment of cerebellar LTD by SOD-catalase or apocynin suggests that ROS is produced in PCs during LTD-inducing stimulation. To test this hypothesis, we next measured intracellular ROS levels of PCs before and after CJS. Because ROS is produced as superoxide and/or hydrogen peroxide (H_2_O_2_) [2,3] and superoxide is very rapidly catalyzed to H_2_O_2_, fluorescent probe for H_2_O_2_ (Amplex Ultrared) was used to monitor intracellular ROS levels [50]. After CJS, fluorescence signals gradually increased in the dendritic region of PCs (Fig. 1F, left panel), suggesting that ROS (superoxide (O_2_^−^) and/or hydrogen peroxide (H_2_O_2_)) production is induced by LTD-inducing stimuli. In contrast, in the presence of apocynin, CJS failed to translate into an increase in the fluorescence signals (Fig. 1F, left and middle panels). These results indicate that ROS (superoxide or hydrogen peroxide) is produced by apocynin-sensitive enzyme(s) during LTD-inducing stimuli.

### 3.4 Impaired long-term adaptation of OKR and cerebellar LTD by inhibition of 8-NO_2_-cGMP signals

Observations of the physiological role of ROS in motor memory and LTD induction in the cerebellum prompted us to investigate the downstream components of ROS. The recent discovery of 8-NO_2_-cGMP, a new component of the nitric oxide (NO)-cGMP pathway, has attracted attention because of its role as a long-lasting cGMP signal in various physiological responses in tissues and cells [54,55]. The generation of 8-NO_2_-cGMP requires ROS, NO, GTP, and guanylate cyclase [55]. 8-NO_2_-cGMP has a similar target as cGMP, including PKG [45]. Furthermore, the ability of 8-NO_2_-cGMP to S-guanylate Cys residues of proteins plays important physiological roles in cell regulation [45]. Although the essential roles of the NO – cGMP – PKG pathway in motor memory and plasticity have been established in the cerebellum [12], the roles of ROS and the 8-NO_2_-cGMP remain unknown.

**Fig. 2:**
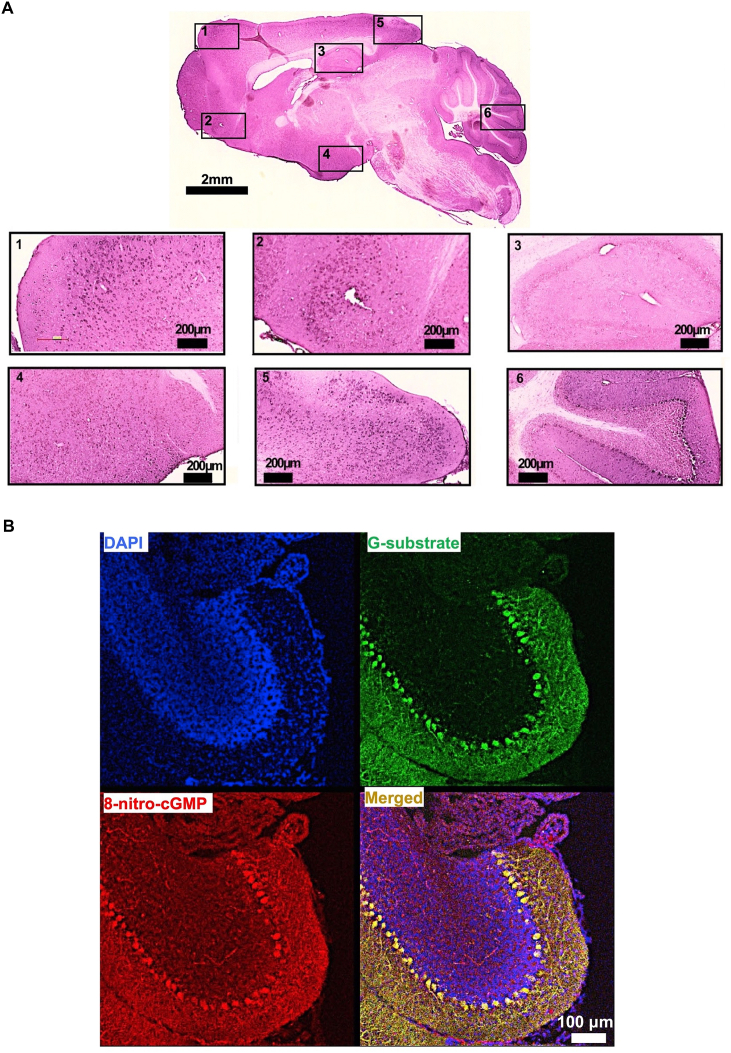
Localization of 8-NO_2_-cGMP in cerebellar Purkinje cells. A, Anatomical distribution of 8-NO_2_-cGMP. Low-power brightfield image showing sagittal histological section (thickness: 20 μm) of mouse brain immunostained with an anti-8-NO_2_-cGMP antibody and counterstained with eosin. Boxed areas (1–6) are enlarged below, showing punctate 8-NO_2_-cGMP staining in cell layers, in particular, cerebellar PC layer (inset number 6). Immunoperoxidase reactions were visualized using DAB as a substrate. Scale bar, 2 mm for whole-brain section; 200 μm for insets. **B,** Confocal images showing distribution of 8-NO_2_-cGMP immunostaining (red) in the mouse cerebellum. Mouse cerebellar sections (thickness: 20 μm) were stained with anti-G-substrate antibody and anti-8-NO_2_-cGMP antibody. Immunoreactivity was visualized using Alexa Fluor 488-labeled (G-substrate: green) and Alexa Fluor 546-labeled (8-NO_2_-cGMP: red) secondary antibodies. Immunostaining for 8-NO_2_-cGMP was co-localized with G-substrate staining in cerebellar PCs. Scale bar, 100 μm. Cell nuclei were stained with 4′, 6-diamidino-2-phenylindole (DAPI; blue). (For interpretation of the references to color in this figure legend, the reader is referred to the Web version of this article.)

We first examined whether 8-NO_2_-cGMP was present in the brain (Fig. 2 and S2A-G). Immunoreactions of 8-NO_2_-cGMP were observed in several brain areas, including the cerebellum (Fig. 2A–B). In mouse cerebellum, 8-NO_2_-cGMP immunostaining appeared to be strongly co-localized with G-substrate, a marker of cerebellar PCs (Fig. 2B). The localization of 8-NO_2_-cGMP in PCs is further validated by its co-localization with calbindin D-28k, another PC maker [56] (Fig. S2E). Notably, 8-NO_2_-cGMP signal, does not co-localize with Neu-N signals associated with granule cells [57] or with GFAP signals indicative of Bergman glia cells (astrocytes, [58]) whose cell bodies are situated in close proximity to PC (Figs. S2A–G). Thus, the high 8-NO_2_-cGMP expression was selectively observed in PCs, a sole output from the cerebellum [12,13,15]. This fact promoted us to examine the role of 8-NO_2_-cGMP in the cerebellum that plays major roles in motor memory [12,13,15].

**Fig. 3:**
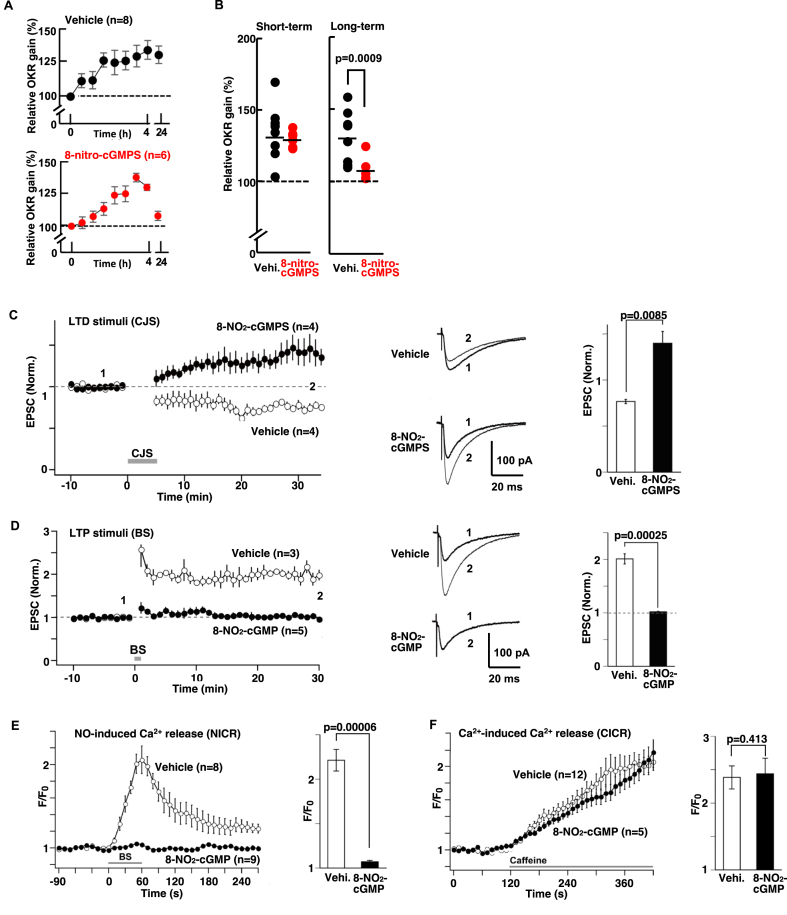
Involvement of 8-NO_2_-cGMP in cerebellar motor learning and LTD induction. **A,** Time course of OKR training and OKR adaptation (4 h, 24 h) in mouse after injection of 8-NO_2_-cGMPS (lower, filled red circles), an inhibitor of 8-NO_2_-cGMP. Repeated measures ANOVA revealed a significant interaction (*F* [1,19] = 15.6, *p* = 0.0009). Data for the vehicle control (upper) are the same control data from Fig. 1B. Thus, statistical analyses were carried out as three groups (vehicle, SOD-catalase, and 8-NO_2_-cGMPS). **B,** Statistical comparison of the relative gain at the end of training (4 h; Short-term) and at 24 h later (Long-term) in the presence of 8-NO_2_-cGMPS (n = 6) or vehicle (n = 8). Same OKR training protocol as before. **C,** Effect of 8-NO_2_-cGMPS on LTD at PF-PC synapses. 8-NO_2_-cGMPS (50 μM) was applied through patch pipette. Left panel: Changes in PF-EPSCs before and after LTD-inducing CJS (LTD stimuli (CJS)) recorded from PCs treated with 8-NO_2_-cGMPS (n = 4) or vehicle (n = 4). Middle panel: Representative traces of PF-EPSCs (1) before and (2) 21–30 min after CJS. Right panel: Average amplitude of PF-EPSCs during 21–30 min after CJS. **D,** Effect of 8-NO_2_-cGMP on LTP at PF synapses. Left panel: Changes in PF-EPSCs before and after LTP-inducing burst stimulation (LTP-stimuli (BS)), recorded from PCs injected with 8-NO_2_-cGMP (n = 5) or the vehicle (n = 3). Middle panel: Representative traces of PF-EPSCs (1) before and (2) after BS. Right panel: Average amplitude of PF-EPSCs during 21–30 min after BS. **E,** Effect of 8-NO_2_-cGMP (50 μM) on NO-induced Ca^2+^ release (NICR). Left panel: Changes in fluorescent signals (Oregon Green 488 BAPTA-1) in PCs treated with 8-NO_2_-cGMP (n = 9) or the vehicle (n = 8) before and after BS. Fluorescent intensity (F) was normalized by corresponding intensity values before BS (F0). Mean ± SEM. Right panel: Peak values of BS-induced NICR in PCs. **F,** Effects of 8-NO_2_-cGMP on Ca^2+^-induced Ca^2+^ release (CICR) elicited by caffeine. Left panel: Changes in fluorescent signals in PCs treated with 8-NO_2_-cGMP (n = 5) or vehicle (n = 12) before and after the application of caffeine (25 mM). Fluorescent intensity (F) was normalized by corresponding intensity values before caffeine application (F0). Right panel: Peak values of caffeine-induced CICR in PCs. Data represent mean ± SEM, except for **B** in which percent relative gains of individual animal are plotted, and horizontal bar indicates mean relative gain. (For interpretation of the references to color in this figure legend, the reader is referred to the Web version of this article.)

We injected 8-nitro-Rp-3′,5′-cyclic monophosphorothioate (8-NO_2_-cGMPS), a competitive inhibitor of 8-NO_2_-cGMP [33], or vehicle into the cerebellar flocculi and subjected the mice to the OKR training/adaptation paradigm as before (Fig. S3A). A significant interaction was found between treatment (8-NO_2_-cGMPS vs. vehicle) and OKR-adaptation phase (short-term vs. long-term; Fig. 3A). At the long-term assessment, OKR adaptation was significantly impaired in the 8-NO_2_-cGMPS group compared to the vehicle control group (Fig. 3B and Fig. S3B; 107.9 ± 3.5 % and 130.8 ± 6.4 % for 8-NO_2_-cGMPS and vehicle, respectively). These results suggest that components of the 8-NO_2_-cGMP-dependent signaling pathway are involved in long-term cerebellar motor memory, but not short-term memory (129.7 ± 2.3 % and 134.3 ± 6.9 % for 8-NO_2_-cGMPS and vehicle, respectively) under the OKR training conditions used.

The possible involvement of 8-NO_2_-cGMP signals in cerebellar motor learning prompted us to examine the involvement of 8-NO_2_-cGMP in cerebellar LTD. Application of 8-NO_2_-cGMPS from a patch pipette to PCs impaired the cerebellar LTD. PF-EPSC amplitudes during the 21–30 min after CJS were 1.40 ± 0.13-fold and 0.766 ± 0.023-fold of the baseline, with or without 8-NO_2_-cGMPS, respectively (Fig. 3C). Taken together, our observations indicated that ROS – 8-NO_2_-cGMP signals are required for the induction of cerebellar LTD and cerebellum-dependent motor memory.

### 3.5 Regulation of immediate early gene (IEG) product in the cerebellar motor learning

8-NO_2_-cGMP is produced from cGMP upon the simultaneous production of NO and ROS [32,33,55]. In the cerebellum, NO is produced by PF activity [49], and ROS by LTD-inducing stimulus (Fig. 1F). Motivated by these findings, we investigated the occurrence of OKR-related neuronal activity in cerebellum, specifically examining the OKR-evoked induction of neuronal activity marker, c-Fos [59]. IEG products show as increase in the cerebellum by eyeblink conditioning, a cerebellum-dependent memory [60,61].

A previous study demonstrated OKR training leads to the significant reduction in synapse number in the middle region of flocculus [62]. They proposed the synaptic elimination induced by the training is a component of neuronal engram for OKR memory. Based on this observation, we analyzed the number of c-Fos-positive PCs in the same area of flocculus (see Figs. S2H–I).

Mice were subjected to 4-h OKR training or no training (sham) as described in Section 2.6 above (see also Figs. S1 and S3 for the training protocol). The average relative OKR gain was 134.6 ± 4.4 % (Mean ± SEM) at the end of the 4-h training. In the absence of OKR training, c-Fos-positive PCs were observed in cell soma and dendrites of PCs (Figs. S2H–I). However, in the flocculus, the number of c-Fos-positive PCs significantly decreased following 4-hr OKR training compared to the sham control mice (Fig. S2H; two-tailed Student's t-test; 17.3 ± 1.57 and 27.4 ± 0.90 for the training and the sham groups, respectively). In the paraflocculus, some c-Fos-positive cells were observed in cerebellum for both OKR-trained and sham mice. However, 4-hr OKR training did not result in a significant alteration for the c-Fos-positive cell number (Fig. S2H; 25.2 ± 2.93 and 28.1 ± 3.18 for the training group and the sham group, respectively). The results demonstrated a decrease in c-Fos, an immediate early gene product, in the floccular PCs by OKR training (Figs. S2H–I), while no significant change was observed in paraflocculus (Figs. S2H–I). Taken together, the reduction of c-Fos-positive PCs by OKR training supports the potential involvement of specific neuronal circuitry activation, including floccular PCs for OKR adaptation.

### 3.6 Inhibition of cerebellar LTP and determination of direction of synaptic plasticity by 8-NO_2_-cGMP

Inhibition of ROS – 8-NO_2_-cGMP signals impaired cerebellar motor learning and cerebellar LTD (Fig. 1, Fig. 3). In particular, upon inhibition of ROS – 8-NO_2_-cGMP signals, CJS elicited LTP-like changes at the PF-PC synapse instead of LTD (Fig. 1D–E and Fig. 3C). Cerebellar LTD is induced by a combination of PF stimulation and depolarization of PCs (CJS) [63], whereas cerebellar LTP is induced only by PF stimulation [49,64,65]. These findings imply that LTP-inducing signals elicited by PF stimulation can also be activated by LTD-inducing stimulation (CJS). Thus, LTD could be abolished by LTP unless the LTP-inducing signal is inhibited, because the amplitude change observed in LTP (more than 100 % potentiation [27,49,65]) is much larger than that of LTD (approximately 20–30 % depression). Because LTP-like change rather than LTD was induced by LTD-inducing stimulus in the presence of inhibitors of ROS or 8-NO_2_-cGMP, we hypothesized that ROS - 8-NO_2_-cGMP signal activated by LTD-inducing stimulus abolish LTP. This hypothesis is partly supported by our previous study, in which bath-applied ROS (H_2_O_2_) abolishes cerebellar LTP [28].

Therefore, in the present study, we further examined whether 8-NO_2_-cGMP inhibits cerebellar LTP. When 8-NO_2_-cGMP was applied from patch pipette to PCs, burst stimulation (BS), which induces LTP in control group, failed to induce LTP. PF-EPSC amplitudes during the 21–30 min after BS were 1.02 ± 0 0.01-fold of baseline levels, which were significantly lower than that of the vehicle control group (2.01 ± 0.10-fold) (Fig. 3D).

NO-induced Ca^2+^ release (NICR) mediated by type 1 ryanodine receptor (RyR1) in the PC is essential for the induction of cerebellar LTP [27]. Therefore, the effect of 8-NO_2_-cGMP on NICR was subsequently examined by Ca^2+^ imaging. With vehicle control, BS resulted in a clear increase in intracellular Ca^2+^ levels (NICR) in PCs (Fig. 3E). In contrast, with 8-NO_2_-cGMP, NICR was greatly diminished. Peak amplitudes in the vehicle control group were 2.21 ± 0.121-fold of the basal level, while in the 8-NO_2_-cGMP group, it was 1.07 ± 0.02-fold (Fig. 3E).

We also examined effects of 8-NO_2_-cGMP on another Ca^2+^ response mediated by RyR1, Ca^2+^-induced Ca^2+^ release (CICR) (Fig. 3F). Caffeine activates this Ca^2+^-dependent Ca^2+^ release. With both vehicle control and 8-NO_2_-cGMP, bath-application of caffeine resulted in a gradual increase in intracellular Ca^2+^ in PCs (2.38 ± 0.17-fold vs. 2.44 ± 0.23-fold, respectively). These results indicated CICR was unaffected by 8-NO_2_-cGMP. Thus, NICR was selectively inhibited by 8-NO_2_-cGMP. Taken together, the ROS–8-NO_2_-cGMP signal plays a role in inhibiting cerebellar LTP through the impairment of NICR and ensures the induction of cerebellar LTD.

### 3.7 Identification of downstream components of 8-NO_2_-cGMP pathway involved in OKR

**Fig. 4:**
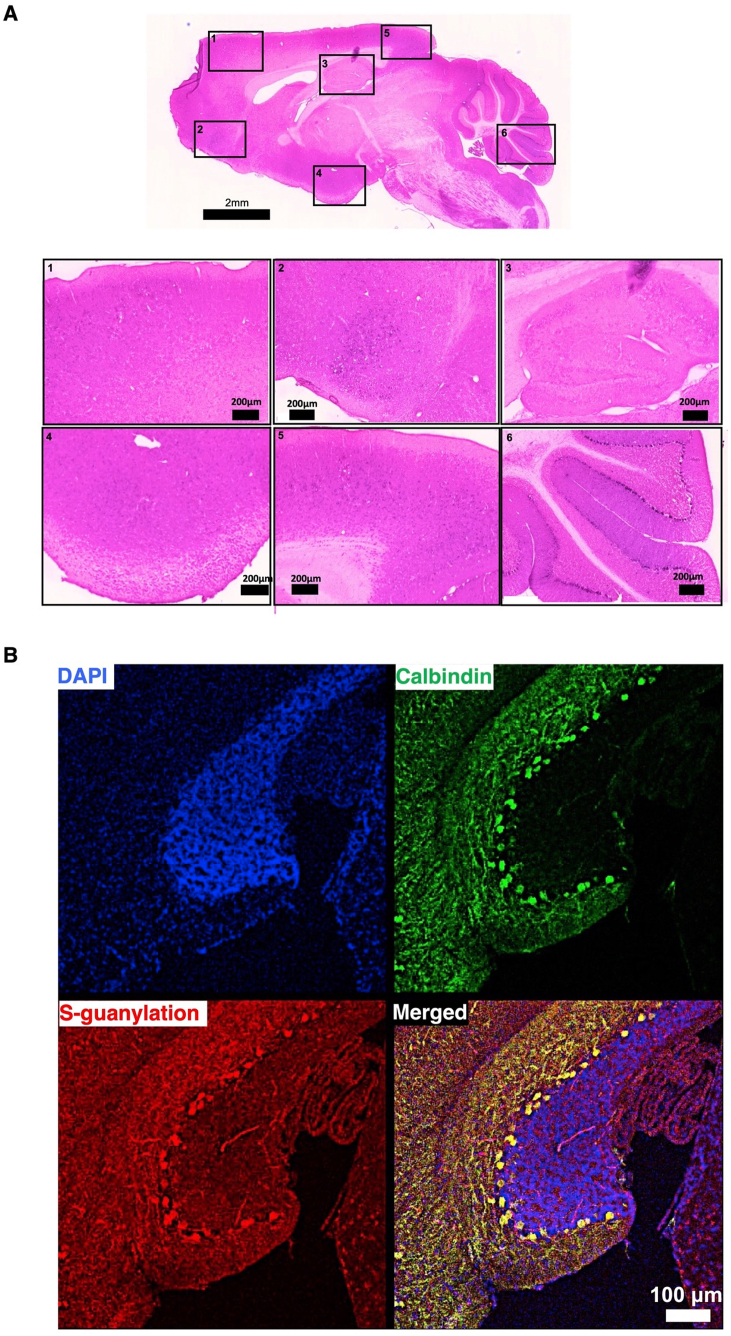
Localization of S-guanylation of proteins in cerebellar PCs. **A,** Low-power brightfield image of sagittal histological brain section (thickness: 20 μm) immunostained with an anti-S-guanylated Cys antibody and counterstained with eosin. Boxed areas (1–6) showing punctate S-guanylated Cys immunoreactivity in PC layer (inset number 6). Immunoreactions were visualized as in Fig. 2A. Scale bar, 2 mm for whole-brain (upper); 200 μm for insets. **B,** Confocal images showing immunostaining of S-guanylated Cys (red) and calbindin (green) in PCs of mouse cerebellum. Immunoreactivity visualized as in Fig. 2B. Scale bar, 100 μm. (For interpretation of the references to color in this figure legend, the reader is referred to the Web version of this article.)

Subsequently, we investigated potential downstream components of 8-NO_2_-cGMP. Modification by 8-NO_2_-cGMP can alter the activity and function of proteins [55]. In the cerebellum, we observed that S-guanylated proteins are concentrated in PCs (Fig. 4A–B). One of the targets of S-guanylation is PKG [45], which is also concentrated in cerebellar PCs [44]. 8-NO_2_-cGMP can induce long-term effects on PKG in two ways: through the S-guanylation of Cys residues in PKG to make it enzymatically active without agonistic binding of cGMP [45], or through its phosphodiesterase (PDE)-resistant agonistic binding to lead the prolonged PKG activation [55].

**Fig. 5:**
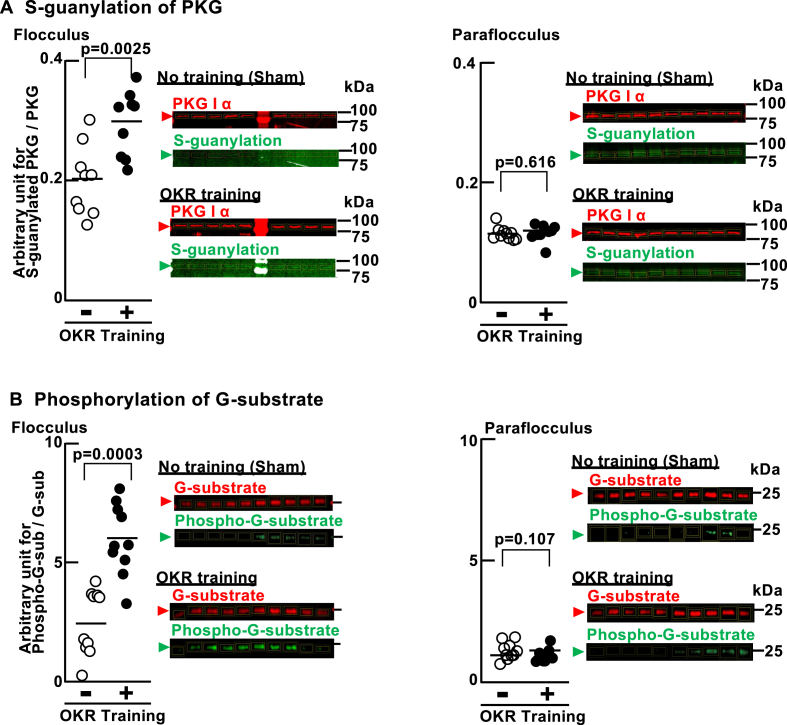
Activation of PKG - G-substrate pathway by cerebellar motor learning. **A,** S-guanylation of PKG1α in flocculi (n = 9) and paraflocculi (n = 10) associated with OKR training (+ symbol in graphs) or sham control training (- symbol in graphs). PKG1α was pulled down using 8-AET-cGMP-gel and analyzed as described in the Methods. Individual data points are plotted, and horizontal lines in graphs indicate group means. For each band in the Western blots (Right), the fluorescence signal for S-guanylated PKG1α (green) was normalized to the signal for total PKG1α (red) and plotted as an arbitrary unit for S-guanylated-PKG/total PKG. One OKR sample was removed from further analysis due to high background in the blot. **B,** Same analysis as in panel A, except analysis performed for phosphorylation of G-substrate in flocculi (n = 10) and paraflocculi (n = 10) induced by OKR training or sham training. The materials unbound to the 8-AET-cGMP gel were processed as described in the Methods and subjected to G-substrate phosphorylation analyses. For each band on the Western blot, the fluorescence signal for phospho-G-substrate was normalized using the signal for total G-substrate and plotted as an arbitrary unit for phospho-G-substrate/total G-substrate. (For interpretation of the references to color in this figure legend, the reader is referred to the Web version of this article.)

We first examined whether OKR training could induce changes in S-guanylation of PKG in the cerebellar flocculi (Fig. 5A and S4A). Mice were subjected to 4-h OKR training or no training (sham), as mentioned in section 2.6 (Figs. S1 and S3). The average relative OKR gain was 131.7 ± 7.4 % (Mean ± SEM) at the end of the 4-h training. Relative ratio of S-guanylated PKG/total PKG increased significantly in the flocculi after this training compared with that in sham control mice (Fig. 5A, left; 0.298 ± 0.03 vs. 0.203 ± 0.03 [arbitrary unit]). The increase appeared to be localized to this part of the cerebellum because S-guanylated PKG remained stable after 4-h OKR training (Fig. 5A, right; 0.116 ± 0.01 vs. 0.119 ± 0.02 [arbitrary unit]) in the adjacent paraflocculi of the same animals (see Fig. 1A).

G-substrate (PPP1R17) is a preferred substrate of PKG [42,43,66] in the cerebellar PCs and its phosphorylation is an indicator of PKG activation in PCs (Fig. 5B and S4B). In the flocculi, the ratio of phospho-G-substrate to total G-substrate was significantly higher for the OKR-training group compared to that of the sham group (6.02 ± 0.47 vs. 2.55 ± 0.43). In the paraflocculi, the ratios of phospho-G-substrate to total G-substrate were statistically indistinguishable for the two groups (1.10 ± 0.09 vs. 1.34 ± 0.11). Taken together, these results indicate that OKR training is associated with the generation of 8-NO_2_-cGMP and modification of PKG and G-substrate in the flocculi.

### 3.8 Inhibition of PDEs by 8-NO_2_-cGMP

Based on its nature as a cyclic nucleotide derivative, we hypothesized that 8-NO_2_-cGMP acts as a PDE inhibitor. Activities of PDEs (PDE1B, 2A1, 3A, 3B, 4D3, 4D7, 5A1, 6C, 7A1, 7B, 8A1, 9A2, 10A1, 10A2, and 11A4) were examined in the presence of 1–10000 nM 8-NO_2_-cGMP (Fig. S5) and specific inhibitors of each PDE. The activities of cGMP-inhibited PDE3A, CaM-stimulated PDE4D3, and cGMP-stimulated PDE7A1 were efficiently inhibited (Fig. S5). Because mRNA expression of these PDEs in the cerebellum has been reported in the Allen Brain Atlas [67], 8-NO_2_-cGMP upon generation may exert PDE inhibitory activities in addition to the S-guanylation and cGMP-like activities. The roles of PDEs in cerebellar-dependent memory and plasticity are largely unknown. However, the finding that 8-NO_2_-cGMP inhibits PDEs suggests that it could potentially prolong the physiological action of cGMP/cAMP in cerebellar PCs to support long-lasting changes required for plasticities and memory. Further analysis of these PDEs will reveal whether the inhibition of PDE by 8-NO_2_-cGMP contributes to cerebellum-dependent memory and plasticity.

### 3.9 Influence of excess amounts of VC and VE on motor coordination and cerebellum-dependent memory

ROS scavengers (antioxidants), such as VC and VE, are believed to be beneficial for scavenging ROS in biological systems; they are commonly and widely used for achievement purposes in athletes and for health promotion purposes in the general public [7]. However, recent reports have demonstrated that excess antioxidants can cause harmful effects such as nulling physical exercise and endurance training in humans [[8], [9], [10]]. Recent reviews have also cautioned on the use of VC/VE because of their inconsistent effects, and VC and VE have been shown to impair training effects [10,11]. Because the essential roles of ROS and their downstream signals in cerebellar motor learning and underlying synaptic plasticity were indicated in the present study (Fig. 1, Fig. 3, Fig. 5), we hypothesized that the harmful effects of VC/VE on physical exercise were from their scavenging functions of physiological ROS generated in the cerebellum.

**Fig. 6:**
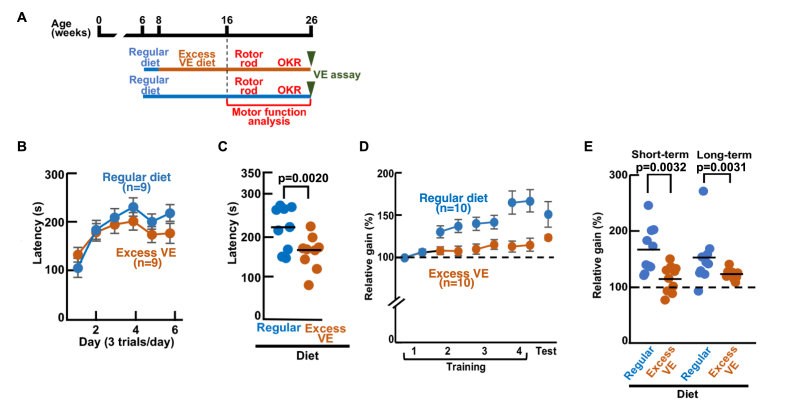
Inhibition of cerebellar motor learning by excessive ingestion of vitamin E. **A,** Schematic diagram showing diet-training-testing schedule for assessing effect of excessive dietary ingestion of vitamin E (VE, α-tocopherol) on motor coordination (rotor rod) and motor memory (OKR adaptation). After the 8-week administration of VE or Regular diet, mice were tested on the rotor rod test, followed by OKR training and adaptation. Quantity of VE in the cerebellum and plasma was measured (see Table S5) at the end of the experiments (26 weeks, motor function analysis group). **B,** Fall latency in the rotor rod test for group ingesting excessive VE (orange filled circles) and group ingesting Regular diet (blue filled circles). Mean ± SEM. **C,** Rotor rod performance for the two diet conditions on the sixth day of the training, with group means indicated by horizontal lines. **D,** Relative gains measured during 4-h OKR training (Training 1–4) and that measured 24 h after the end of 4-h training (Test) for the two diet conditions (Refer to Fig. S6B for the training protocol). Mean ± SEM. **E,** Relative gains at the end of the 4-h training (Short-term) and that measured at 24 h after the training (Long-term) with horizontal lines indicating group means. (For interpretation of the references to color in this figure legend, the reader is referred to the Web version of this article.)

To test this hypothesis, we examined the effects of VC/VE overdose on cerebellar motor learning. Excess VC and VE were administered systemically to mice through diet and drinking water for 8 weeks (Fig. 6A–E) to mimic the chronic intake of VC/VE through supplements. In the present experiment, we mimicked supplement intake conditions, administering submaximal amounts of VC and VE orally without affecting drinking and eating behaviors [28,37,38]. In the VC/VE assay group (Table S4), following the administration of VC/VE for 8 weeks, the VE content in the cerebellum was significantly higher in the excess VC/VEgroup (11.2 ± 1.1 μg/mg tissue for the Regular diet group and 13.0 ± 1.2 μg/mg tissue for the Excess VC/VE group). After the 18-week administration, the contents of VE were again significantly higher in the Excess VC/VE group (16.4 ± 2.7 μg/mg tissue) than in the Regular diet group (13.4 ± 0.9 μg/mg tissue). However, the contents of VC stayed relatively stable in 8-week administration (3.2 ± 0.3 and 3.4 ± 0.3 μg/mg tissue for the Regular diet and Excess VC/VE groups, respectively; Student's t-test) and 18-week administration (3.6 ± 0.2 and 3.7 ± 0.3 μg/mg tissue for the Regular diet and Excess VC/VE groups, respectively).

Then, we examined the cerebellum-dependent behavior in these mice (Figs. S6A–B). Excess VC/VE administration disturbed the motor function in the rotor rod test (Figs. S6C–D). The performance of the Excess VC/VE group did not reach that of the Regular diet group on sixth day of the training. These results indicated that Excess VC/VE disturbed motor learning in mice, especially in the latter half of the 6-day training period. Next, we examined the influence of Excess VC/VE on the OKR of eye movement (Figs. S6E–F). The OKR gain increased gradually during the 4-h OKR training, and short-term adaptation (short-term memory) was measured at the end of the 4-h training period. Then, long-term adaptation was measured at 24 h after the end of 4-h training (Figs. S6E–F). Short-term adaptation showed no significant difference between the Regular diet and Excess VC/VE groups (166.9 ± 12.5 for the Regular diet group and 149.2 ± 8.2 for the Excess VC/VE group). The long-term adaptation was significantly reduced in the Excess VC/VE group (159.9 ± 8.0 for the Regular diet group and 131.4 ± 6.1 for the Excess VC/VE group). These results indicated that Excess VC/VE administration specifically impairs long-term adaptation in OKR.

The disturbed motor memory caused by the combination of VC/VE administration led us to examine the influence of VE and VC separately. After the 8-week administration of a diet containing either excess VE or VC to the mice (Fig. 6A and S6G), the rotor rod test and OKR were examined (Fig. 6B–E and S6H–K). In the cerebellum, the Excess VE administration for 8 weeks led to significantly higher VE in the Excess VE group (12.7 ± 0.4 μg/g tissue for the Regular diet group and 13.8 ± 0.4 μg/mg tissue for the Excess VE group) (Table S5). Administration of Excess VE alone disturbed the performance of the rotor rod (Fig. 6B–C). On the sixth day of the training, the latencies to stay on the rod were 219.4 ± 17.2 s for the Regular diet group and 162.7 ± 13.6 s for the Excess VE group (Fig. 6C). Short-term OKR adaptation was significantly impaired (Fig. 6D–E; 166.9 ± 13.1 % for the Regular diet group and 115.2 ± 7.7 % for the Excess VE group). Long-term adaptation was also impaired (153.9 ± 14.1 % for the Regular diet group and 120.6 ± 2.2 % for the Excess VE diet group).

In the cerebellum, the VC contents were 4.0 ± 0.1 μg/mg tissue for the Regular diet group and 4.3 ± 0.1 μg/mg tissue for the Excess VC group after 8-week administration (Table S5). Based on our previous report [32], the administration of current Excess VC diet is equivalent to 1000 mg/day VC for human weighing 60 kg. This dosage exceeds the recommended daily allowance for VC recommended by FDA (90 mg for male and 75 mg for female at age of 19 years old and above).

However, Excess VC administration (Fig. S6G) did not influence the rotor rod performance, even on the sixth day (Figs. S6H–I; 264.0 ± 14.7 s for the Regular diet group and 233.5 ± 14.8 s for the Excess VC group). OKR performance was also not influenced by Excess VC. The short-term OKR adaptation was 119.7 ± 4.4 % and 126.6 ± 6.9 % for the Regular and Excess VC groups, respectively (Figs. S6J–K). The long-term adaptation was 118.9 ± 4.0 % and 120.9 ± 4.1 % for the Regular and Excess VC groups, respectively (Figs. S6J–K).

One of the concerns regarding the use of an additional component in the diet, such as VC, is its potential impact on the diet consumption by mice. It is possible that reduced caloric consumption may contribute to the *in vivo* reduction of ROS (for review, see Ref. [68]). To investigate this possibility, we conducted measurements on body weight and diet consumption, as outlined in Table S6. The mice body weight before the administration was 22.7 ± 0.2 and 22.6 ± 0.3 g for the Regular diet and Excess VC diet groups, respectively (two-tailed Student's t-test). After 8-week of the administration, no significant difference was observed in body weight (28.7 ± 0.5 and 28.8 ± 0.4 g for the Regular diet and Excess VC diet groups, respectively; two-tailed Student's t-test). Both Regular diet group and Excess diet group gained weight similarly following the 8-week administration (22.7 ± 0.2 to 28.7 ± 0.5 g for Regular diet; 22.7 ± 0.3 to 28.8 ± 0.4 g for Excess VC diet). This increase possibly reflects the growth of mice in 8 weeks.

No apparent difference in daily diet consumption/cage (housing 5 mice) was observed between Regular diet group and Excess VC diet group (Table S6) though the measurement of daily consumption in each mouse was not carried out in this study. These results may support the idea that Excess VC diet may not alter the diet consumption and the metabolism in the mice.

Further experiments are necessary to gain a deeper understanding of the metabolic changes induced by the Excess VC diet and to elucidate the precise mechanisms underlying the physiological roles of ROS.

## 4 Discussion

The involvement of endogenous ROS – 8-NO_2_-cGMP signals in cerebellar motor learning (OKR) and LTD was revealed (Fig. 1, Fig. 2, Fig. 3, Fig. 4). Although the effects of exogenous ROS on synaptic plasticity have been investigated in various brain regions [69,70], the involvement of endogenous ROS, produced by physiological neuronal activity, has not been demonstrated. This study is the first to demonstrate the involvement of endogenous ROS in synaptic plasticity.

In the present study, we observed the high expression of 8-NO_2_-cGMP (Fig. 2 and S2), the OKR-dependent increase in S-guanylation of PKG (Fig. 5A) and the phosphorylation of G-substrate (Fig. 5B) that is expressed in PCs in the cerebellum. Based on these findings, we propose that the ROS – 8-NO_2_-cGMP pathway is activated in an OKR-dependent manner in the cerebellum. To further substantiate activation of this signaling pathway by neuronal activity during OKR training, we examined changes in the expression of c-Fos, an immediate early gene (IEG) product, in floccular PCs subjected to OKR training. Rapid and selective upregulation of IEGs including c-Fos occurs in subsets of neurons within specific brain regions is associated with learning and memory formation (for recent review, see Ref. [71]). Calcium influx is crucial for inducing IEG expression (for recent review, see Ref. [66]).

Surprisingly, clear signals in non-trained groups and the significant decrease after the OKR training were observed. This was unexpected, considering that Ca^2+^ signals in PCs are elevated by neuronal activity inducing LTD, the cellular basis for OKR [72]. It is conceivable that some factor(s) activated by neuronal activity during OKR training inhibits increase in c-Fos expression in PCs.

Additionally, LTD at PF synapse is a cellular basis for OKR [51] and number of AMPA-type glutamate receptor and spine are decreased at PF synapse by OKR [62]. Hence, it is also plausible that PF-mediated Ca^2+^ release in PCs, such as IP3-induced Ca^2+^ release [73,74], and/or NO-induced Ca^2+^ release [27], are decreased by OKR training, leading to the decrease in c-Fos expression level in PCs. The precise molecular mechanism might be elucidated by further studies.

Impairment of LTD at PF-PC synapse by apocynin, an inhibitor of ROS-producing enzymes, including NOX and DUOX, suggests their possible involvement in LTD at PF-PC synapse. NOX4 and 5 as well as DUOX1 and 2 are Ca^2+^-dependent (activatable) ROS synthases [3,52]. Because LTD-inducing stimuli evoke a large Ca^2+^ transient (surge) in cerebellar PCs [72,75], Ca^2+^-activated NOX and DUOX could play a role for LTD at PF-PC induction. In the current study, we employed pharmacological interventions to investigate the role of the ROS – 8-NO_2_-cGMP pathway. However, it is important to acknowledge that these interventions can sometimes cause unwanted side effects especially *in vivo*, potentially leading to misleading results. The electrophysiological and behavioral analysis of PC-specific KO mice for ROS generating enzymes including NOX and DUOX will reveal the ROS generation of these enzymes in PCs. Additionally, analyzing knock-in mice that carry PKG1α with a conversion of the S-guanylation site cysteine to alanine will help confirm the involvement of PKG and its specific amino acid residues in neuronal plasticity and memory within the cerebellum.

In our excess ascorbic acid (VC)/vitamin E (VE) experiments (Fig. 6 and S6), we attempted to mimic the amounts of antioxidant supplements commonly used by athletes and the general public (for a comprehensive review, please refer to Refs. [[9], [10], [11]]). However, to fully understand the effect of VC/VE on cerebellum-dependent memory, it is preferable to establish a dose-response curve though the present experiment mimicked supplement intake conditions, administering submaximal amounts of VC and VE orally without affecting drinking and eating of mice behaviors [28,37,38]. To find the influence of the higher VC concentrations on cerebellum-dependent behaviors is difficult to achieve in oral administration. For the systemic administration of VE that has various physiological functions, it is plausible that it could cause motor impairment by affecting not only the cerebellum but also other areas of the brain, muscles, and peripheral organs (for further insights, please refer to Ref. [76]). Therefore, additional analyses assessing general motor functions are required to accurately interpret our current results.

Furthermore, we employed VC/VE as ROS scavengers for long-term administration prior to the rotor rod test and OKR analyses, and injected SOD/catalase into the cerebellar flocculus during the OKR experiments to scavenge superoxide and hydrogen peroxide. It is also important to note that additional experiments utilizing specific inhibitors of NOX and DUOX, instead of VC/VE, may elucidate the specific components of ROS involved in memory function *in vivo*. Although we demonstrated that the ROS-specific scavengers, SOD and catalase, inhibit the OKR in the cerebellar flocculus (Fig. 1), conducting additional experiments involving the systemic administration of non-toxic ROS scavengers suitable for long-term oral consumption will provide further clarity on whether the VC/VE effects observed in the present research are reliant on ROS.

In our previous study, we demonstrated that application of ROS (100 μM H_2_O_2_) on cerebellar slices did not induce LTP nor LTD [28]. The finding is consistent with previous studies that demonstrated the involvement of a variety of signaling molecules besides ROS – 8-NO_2_-cGMP [12,13]. Thus, we concluded that the ROS – 8-NO_2_-cGMP signal is essential for cerebellar LTD induction, however, the additional signal is also required to induce LTD.

The physiological roles of ROS and 8-NO_2_-cGMP in behavioral, electrophysiological, and molecular aspects support previous reports of impairments of motor-related exercise and/or functions by antioxidant supplements [[8], [9], [10], [11]]. Antioxidant supplements potentially diminish the signal-conveying ROS necessary to improve athletes’ performance at the level of synapse and cerebellar network (Fig. 1, Fig. 2, Fig. 3, Fig. 4, Fig. 5, Fig. 6) that controls skilled learning [12]. Our results may partly explain the contradictory effects of antioxidant supplements in exercise [10,11], which involve a variety of muscle coordination rather than a pair of lateral rectus muscles for horizontal OKR. Notably, ROS and 8-NO_2_-cGMP are involved in long-term memory, although the results in short-term memory are to be elucidated. Furthermore, we demonstrated that OKR training induced a significant increase in G-substrate phosphorylation (a PKG substrate) in cerebellar PCs. Together with our previous results on the specific involvement of G-substrate in long-term memory [36], the current results suggest the essential roles of the 8-NO_2_-cGMP – PKG – G-substrate pathway as a molecular mechanism underlying long-term memory in the cerebellum.

Cerebellar PF-PC synapses show bidirectional synaptic plasticity, LTD and LTP [51,77]. BCM theory has been proposed for direction determination (potentiation/depression) [78]. The theory hypothesizes the essential roles of intracellular Ca^2+^ levels in determining the direction of synaptic plasticity: a high Ca^2+^ threshold for potentiation (LTP) and a low Ca^2+^ threshold for depression (LTD) in forebrain synapses. The signals for both LTP and LTD are speculated to be activated at higher Ca^2+^ levels. The apparent direction of plasticity is determined by the summation (or addition) of LTP and LTD, so that LTP is observed at high Ca^2+^, even though both LTP and LTD signals are activated in the same cell.

**Fig. 7:**
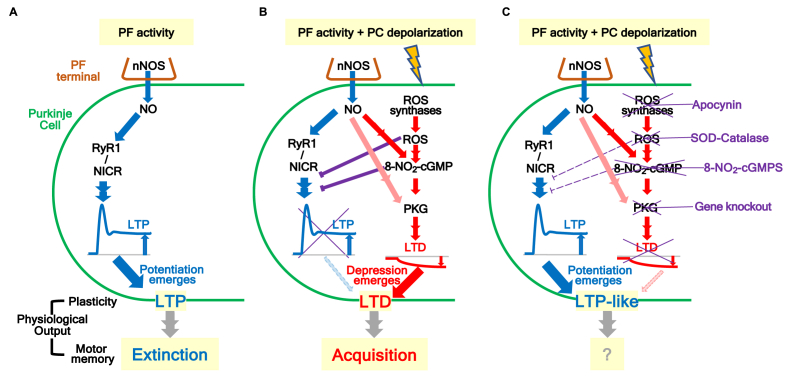
Possible signaling mechanisms of cerebellar synaptic plasticity and motor memory. **A**, Repeated stimulation of parallel fibers (PF) alone induces NO production leading to low Ca^2+^ elevation through NO-induced Ca^2+^ release mediated by RyR1. Activation of the RyR1 (by NO) and protein phosphatases (PPs; not shown in this Figure) induces LTP [49,64,65], which is theoretically involved in the extinction of cerebellar motor learning [17]. **B**, When PF stimulation is synchronized with depolarization of PCs, ROS is produced in addition to NO. Coincident production of NO and ROS activates 8-NO_2_ – cGMP – cGMP-dependent protein kinase (PKG) signals and induces LTD, the cellular basis for cerebellar motor learning. Although LTD cannot surpass LTP, ROS [28] and 8-NO_2_-cGMP abolish LTP-inducing signals to ensure the induction of LTD. **C**, In the presence of SOD-catalase, apocynin or 8-NO_2_-cGMPS, ROS, ROS synthases (NOX and DUOX) or 8-NO_2_-cGMP are inhibited, respectively. Therefore, LTD induction as well as LTP inhibition are abolished, and residual LTP is observed. On the other hand, in the cerebellum of PC-specific PKG-ablation mice (Gene knockout), LTD induction is impaired, and LTP-like change is not induced by LTD-inducing stimulus [80].

On the other hand, in the cerebellum, LTP is induced at PF-PC synapse, when PF alone is stimulated (Fig. 7A), and LTD is induced when the PF stimulation is synchronized with PC depolarization (Fig. 7B). Because PC depolarization results in larger intracellular Ca^2+^ transient, it is speculated that a high Ca^2+^ threshold for LTD and a low Ca^2+^ threshold for LTP at PF-PC synapses [79]. However, the LTD-inducing stimulus activates LTP-inducing signals as well as LTD-inducing signals, it is hypothesized that some signals activated by LTD-inducing stimulus inhibit LTP signals so that LTD can be induced. We previously demonstrated that ROS inhibited LTP at PF-PC synapse [28]. In current study, we showed that LTP was inhibited by 8-NO_2_-cGMP (Fig. 3). In addition, we revealed that ROS was generated by LTD-inducing neuronal activity (Fig. 1), followed by 8-NO_2_-cGMP production, which requires ROS, via cerebellar motor learning (Fig. 5). These observations indicate that the ROS – 8-NO_2_-cGMP signal potentially inhibits LTP induction, leading to LTD during cerebellar motor learning/LTD-inducing activity (Fig. 7B). In contrast, in the presence of SOD-catalase, apocynin or 8-NO_2_-cGMPS, LTD signal is impaired whereas LTP-inducing signals is released from the inhibition by ROS or 8-NO_2_-cGMP. As a result, LTP-like plasticity is induced by LTD-inducing stimulus (Fig. 7C, see also Fig. 1, Fig. 3C).

A possible mechanism for the inhibition of cerebellar LTP by 8-NO_2_-cGMP is modulation of Ca^2+^ release, specifically, inhibition of NICR (Fig. 3E). NICR is a novel Ca^2+^ release mechanism first identified in cerebellar PCs and essential for induction of PF-LTP [27]. S-nitrosylation of RyR1 is essential for the induction of NICR [27,81]. Thiol residues in cysteine in RyR1 are targets of redox modifications, including S-nitrosylation by NO, disulfide formation by ROS, and S-guanylation by 8-NO_2_-cGMP. We previously demonstrated that ROS inhibited protein S-nitrosylation in the cerebellum [28]. This finding suggests that endogenous 8-NO_2_-cGMP, as with ROS, inhibits S-nitrosylation of cerebellar RyR1, which may lead to reduction of NICR activity.

Another possible role of 8-NO_2_-cGMP is through the protein phosphatases (PPs) inhibition. PP1, PP2A, and PP2B are involved in cerebellar LTP [29,82], and 8-NO_2_-cGMP activates PKG to phosphorylate G-substrate [83] (Fig. 5B). The inhibition of PP1/2A by phospho-G-substrates [34,83] suggests that 8-NO_2_-cGMP potentially abolishes PF-LTP by inhibiting PP1 and/or PP2A.

G-substrate phosphorylation in the cerebellar flocculi increased after OKR training (Fig. 5B). The results are consistent with previous observations of G-substrate phosphorylation in response to LTD-inducing stimuli such as the application of high-K and glutamate to mimic neuronal activity [34]. The inhibition of PDEs by 8-NO_2_-cGMP (Fig. S5) may also contribute to PKG-dependent phosphorylation of G-substrate (Fig. 5B). The PKG – G-substrate pathway may play a pivotal role in the PKG activation by cGMP and 8-NO_2_-cGMP, leading to enhanced protein phosphorylation through the inhibition of PP by phosphorylated-G-substrate.

ROS has long been considered harmful to our body since Denham Harman proposed that ROS contribute to aging and diseases [1]. However, it is important to note that ROS generation occurs in an active and signal-dependent manner [3]. Notably, various brain regions, including the hippocampus, exhibit the presence of 8-NO_2_-cGMP and S-guanylated proteins (see Fig. 2, Fig. 4). Previously, we conducted experiments where we observed increased S-guanylation of hippocampal proteins by intracerebroventricular infusion of 8-NO_2_-cGMP in mice [84]. However, it is worth noting that the levels of 8-NO_2_-cGMP and S-guanylated proteins in the hippocampus were lower compared to those in the cerebellum (also refer to Fig. 2, Fig. 4). The infusion of 8-NO_2_-cGMP resulted in an increase in S-guanylation of SNARE-25 and formation of the SNARE complex, which may influence neurotransmitter release and subsequently affect neuronal activity and hippocampal functions including memory processes. Interestingly, the infusion of 8-NO_2_-cGMP led to a reduction in hippocampus-dependent fear memory [84]. The observed increase in c-Fos-positive cells in the hippocampus may indicate enhanced neuronal activity during fear memory-inducing training. It is possible that 8-NO_2_-cGMP in the hippocampus could directly or indirectly contribute to memory impairment, possibly through its influence on the neuronal network, considering the intracerebroventricular delivery of the infusion we have used. Further investigation may shed light on the potential involvement of the ROS – 8-NO_2_-cGMP pathway in the hippocampus, a prominent site associated with declarative memory.

In general, long-term memory requires long-lasting molecular mechanisms that sustain plasticities and gene regulation [85]. For example, calmodulin-dependent protein kinase II (CaMKII) is a candidate for long-acting mechanisms of neuronal plasticity and memory in the hippocampus [86] (for recent review, [87]). However, in the cerebellum, the specific molecules and mechanisms responsible for long-lasting neuronal plasticity and long-term memory remain unknown. In this study, we demonstrated that ROS scavengers (SOD-catalase) and an inhibitor of 8-NO_2_-cGMP (8-NO_2_-cGMPS) did not influence short-term memory in OKR adaptation, but they did compromise long-term memory (Fig. 1, Fig. 3). Moreover, we previously observed impairment of long-term memory and intact short-term memory in G-substrate KO mice [36]. Hence, in addition to the crucial roles of cGMP–PKG in cerebellar LTD and eye movement adaptation as suggested by PKG-ablated mice [77], the S-guanylated PKG produced by 8-NO_2_-cGMP through ROS emerges as a promising molecular candidate underlying long-term memory in the cerebellum. Collectively, the ROS – 8-NO_2_-cGMP – PKG – G-substrate pathway reported here is indicated to be a good candidate for a long-lasting mechanism to control memory. How this translates into the organization and activity of the cerebellar-cortical circuitry that enables animals to remember and execute learned movements over the course of their lives [12] will be clarified by future studies.

## Funding

This study was supported in part by grants from JSPS, Japan (19K22834, 21K19752, 23K18456 [SE, SK], 18K08878 and 19H04044 [SE], 19K06955 and 23K06012 [SK]; 21H05258, 21H05263, 22K19397 and 23K20040 [TAk]); JST CREST, Japan JPMJCR2024 [TAk]; AMED, Japan JP21zf0127001 [TAk]; Shimizu Foundation for Immunology and Neuroscience Grant, Japan [SK]; Brain Science Foundation, Japan [SK].

## Data and materials availability

All study data are included in this article and Supplementary material.

## CRediT authorship contribution statement

**Sho Kakizawa:** Conceptualization, Data curation, Formal analysis, Funding acquisition, Investigation, Methodology, Resources, Validation, Visualization, Writing – original draft, Writing – review & editing. **Tomoko Arasaki:** Conceptualization, Data curation, Formal analysis, Investigation, Methodology, Validation, Visualization, Writing – original draft, Writing – review & editing, Resources. **Ayano Yoshida:** Data curation, Formal analysis, Investigation, Methodology, Writing – original draft. **Ayami Sato:** Data curation, Formal analysis, Investigation, Methodology, Validation. **Yuka Takino:** Data curation, Formal analysis, Investigation, Methodology, Validation. **Akihito Ishigami:** Conceptualization, Data curation, Formal analysis, Investigation, Methodology, Validation, Visualization, Writing – original draft. **Takaaki Akaike:** Data curation, Formal analysis, Investigation, Methodology, Validation, Visualization, Writing – original draft, Resources. **Shuichi Yanai:** Conceptualization, Data curation, Formal analysis, Investigation, Methodology, Validation, Visualization, Writing – original draft, Writing – review & editing. **Shogo Endo:** Conceptualization, Data curation, Formal analysis, Funding acquisition, Investigation, Methodology, Project administration, Resources, Supervision, Validation, Visualization, Writing – original draft, Writing – review & editing.

## Declaration of competing interest

All authors declare no potential conflict of interest in the above paper.

## Data Availability

No data was used for the research described in the article.
