# Supplementary material for: Essential role of ROS – 8-Nitro-cGMP signaling in long-term memory of motor learning and cerebellar synaptic plasticity
Source: Redox Biol. 2024 Feb 1;70:103053. doi: 10.1016/j.redox.2024.103053 (PMC10869263; doi:10.1016/j.redox.2024.103053)
Supplement: Multimedia component 1 [file mmc1.pdf]

Supplementary Materials for

Essential Role of ROS – 8-nitro-cGMP signaling in long-term memory of motor learning and cerebellar synaptic plasticity

Sho Kakizawa[a,*,1], Tomoko Arasaki[b,1], Ayano Yoshida[b], Ayami Sato[c], Yuka Takino[c], Akihito Ishigami[c], Takaaki Akaike[d], Shuichi Yanai[b], and Shogo Endo[b,*]

[a]Department of Biological Chemistry, Graduate School and Faculty of Pharmaceutical Sciences, Kyoto University, Kyoto 606-8501, Japan

[b]Aging Neuroscience Research Team, [c]Molecular Regulation of Aging, Tokyo Metropolitan Institute for Geriatrics and Gerontology, Tokyo 173-0015, Japan

[d]Department of Environmental Medicine and Molecular Toxicology, Tohoku University Graduate School of Medicine, Sendai 980-8575, Japan

[*]Correspondence: kakizawa.sho.4u@kyoto-u.ac.jp (S.K.) or sendo@tmig.or.jp (S.E.)

(Lead contact: Dr. Shogo Endo sendo@tmig.or.jp)

[1]Equally contributed to the work.

This file includes: Figs. S1-S6 and Tables S1-S6

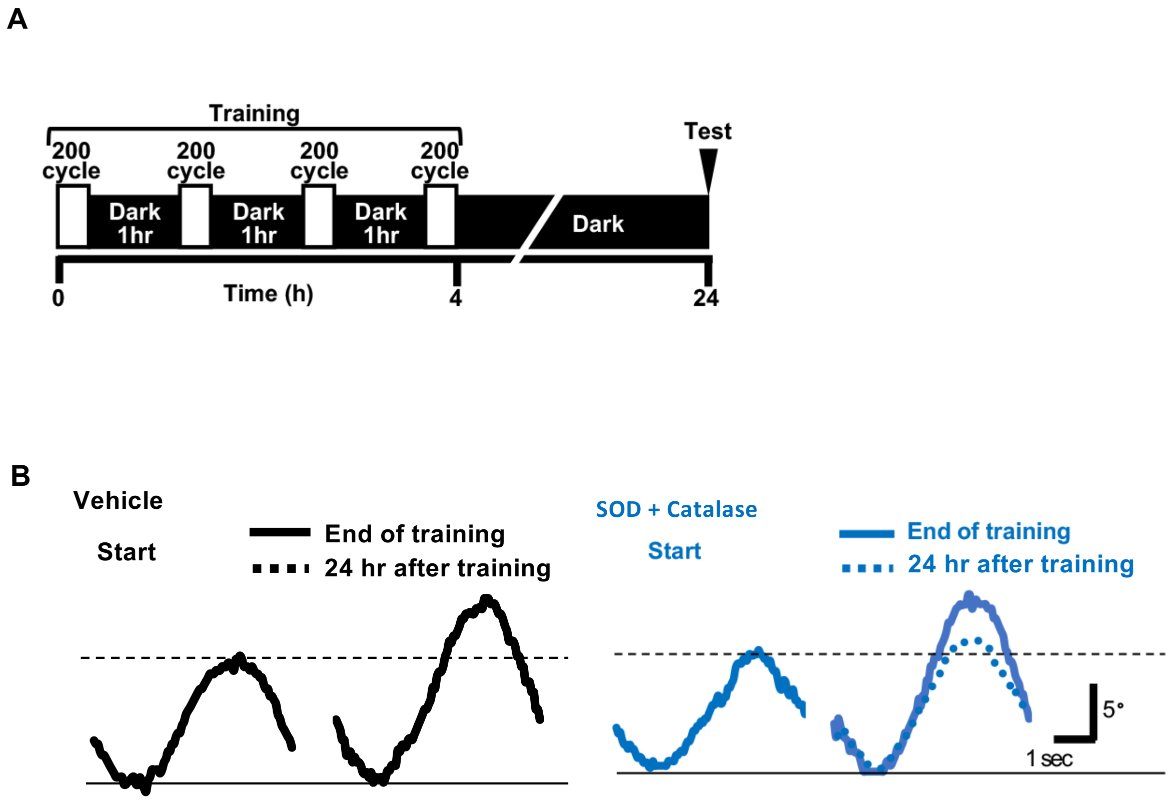

Fig. S1. Involvement of activity-dependent ROS induction in cerebellar learning and plasticity.

(A) Training protocol for optokinetic response (OKR) and OKR adaptation in mice. OKR training consisted of presenting to the mice four sets of 200 cycles of sustained sinusoidal screen oscillations at 15° and 0.17 Hz; the mice had a 1-hr rest period in the dark between set presentations. Short-term OKR adaptation was measured at 24 hr after the end of the 4-hr training. Mice were kept in the dark in their cages after training. Long-term OKR adaptation was then assessed at 24 hr after the end of 4-hr training.

(B) Sample trace of OKR before and 24 hr after the 4-hr training in saline vehicle- (control) and SOD-catalase-injected mice.

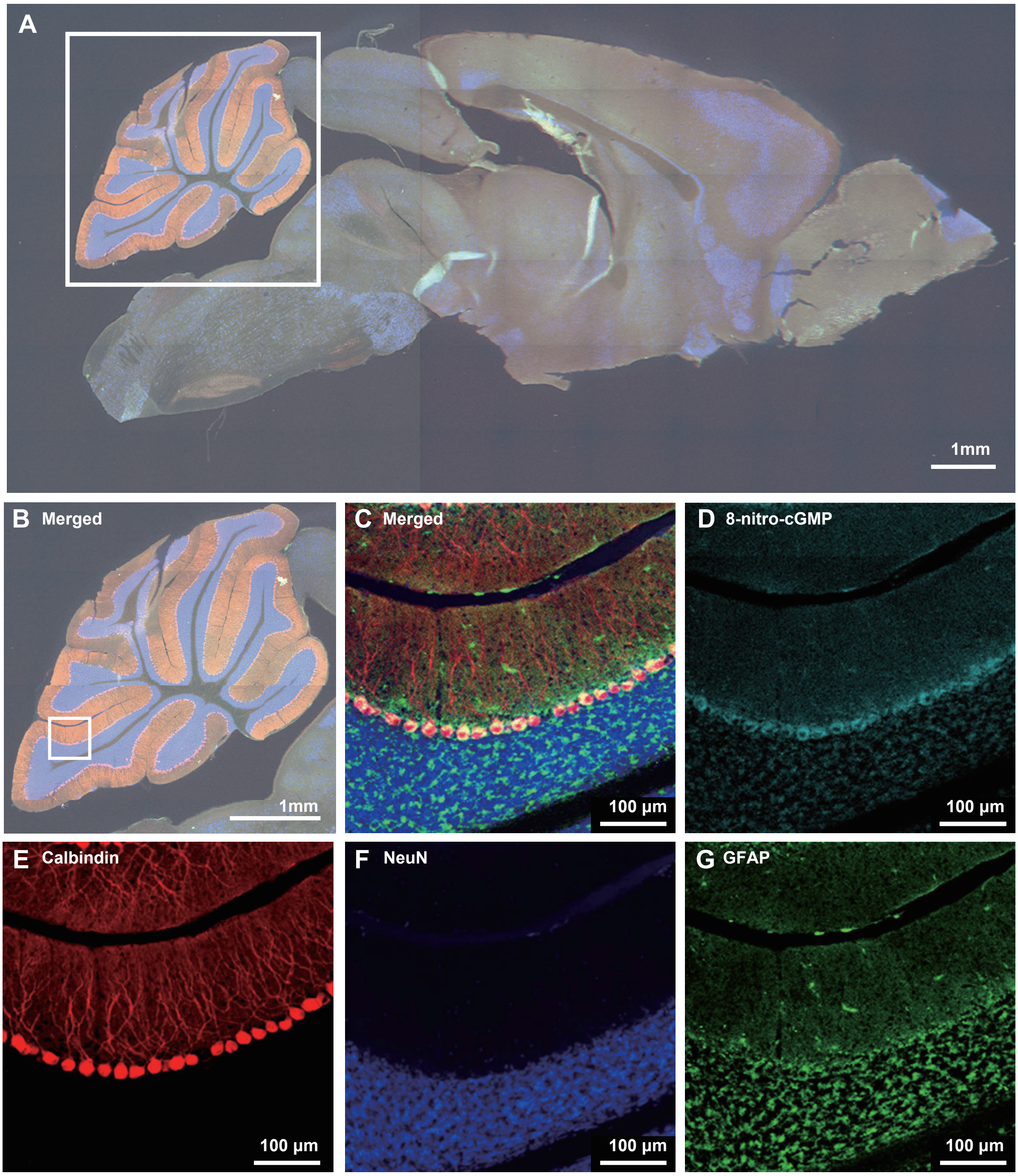

A
1mm
B Merged
1mm
C Merged
100 µm
D 8-nitro-cGMP
100 µm
E Calbindin
100 µm
F NeuN
100 µm
G GFAP
100 µm

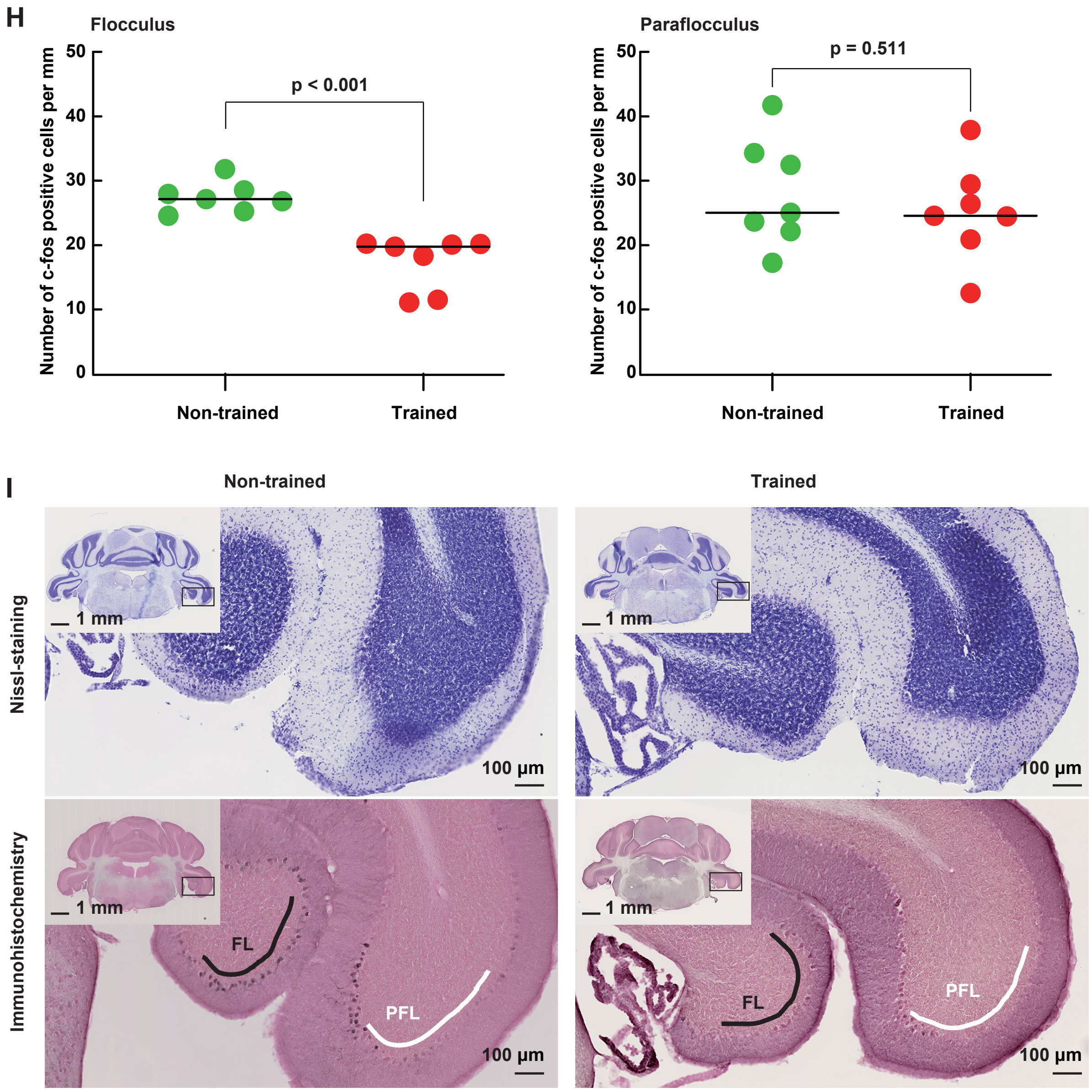

Fig. S2. (A)-(G), immunofluorescence staining of mouse brain slice (30 µm) for 8-nitro-cGMP with cell markers. (A)-(C) were merged confocal fluorescent picture. **(D)** antibodies against mouse 8-NO_2-cGMP with Alexa Fluor 405-labeled anti-mouse IgG, **(E)** Alexa Fluor 594-labeled anti-calbindin antibody (Purkinje cells; PCs), **(F)** Alexa Fluor 647-labeled anti-NeuN (granule cells), **(G)** Alexa Fluor 488-labeled anti-GFAP antibody (glia). The boxed cerebellar area in (A) is enlarged in (B) and further enlarged in (C). Scale bars, 1mm for (A) and (B); 100 µm for (C)-(G).

(H) and (I), analysis of c-Fos expression in cerebellar Purkinje cells following 4-hr optokinetic response (OKR) training. Mice subjected to 4-hr OKR training (Trained) and sham-treated mice (Non-trained) were evaluated for c-Fos-positive cells in cerebellar slices using an anti-c-Fos antibody. c-Fos-positive PCs in the flocculus (marked by the black line) and paraflocculus

(marked by the white line) were quantified. The number of c-Fos-positive cells was normalized by the length of the PC layer (black and white lines). Enlarged views of the areas containing the flocculus and paraflocculus are depicted in the insets. Scale bar, 1 mm for insets and 100 μm for enlarged flocculus and paraflocculus.

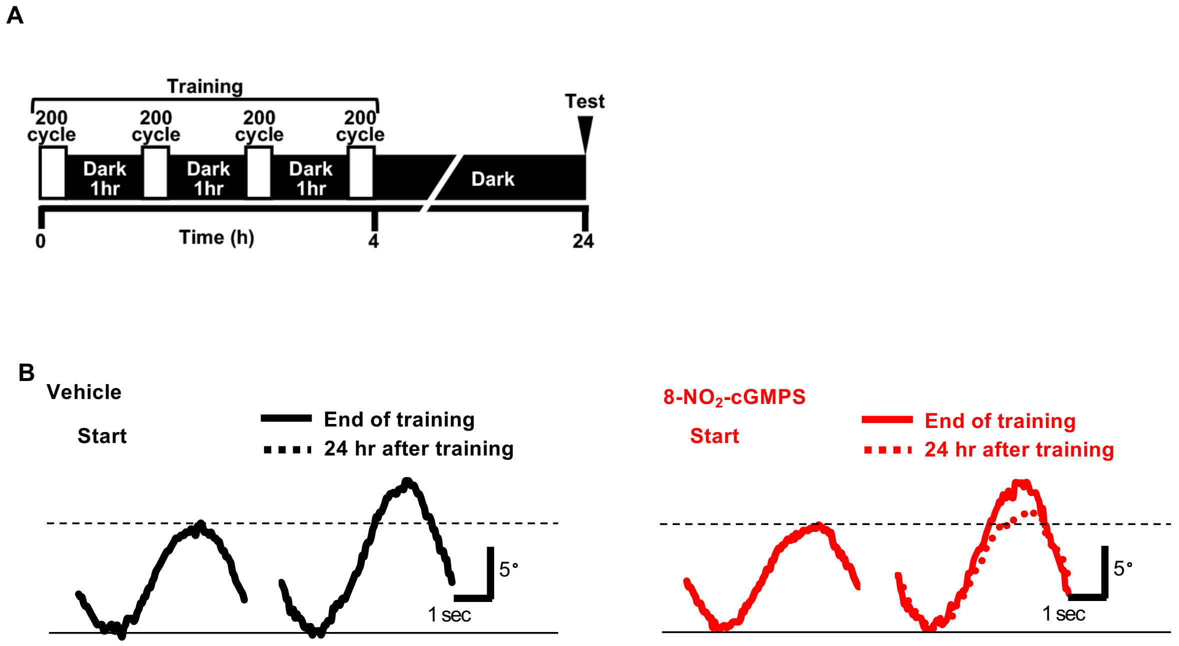

Fig. S3. Involvement of 8-NO$_2$-cGMP in cerebellar learning.

(A) Training protocol for OKR. OKR training consisted of presenting four sets of 200 cycles of sustained sinusoidal screen oscillations at 15° and 0.17 Hz with a 1 hr rest period in the dark between the set presentations. Short-term OKR adaptation was measured at the end of the 4-hr training. Mice were kept in the dark in their cages after training. Long-term OKR adaptation was then assessed 24 hr after the end of the 4-hr training.

(B) Sample trace of OKR before and 24 hr after the 4-hr training in saline vehicle- (control) and 8-nitro-cGMPS-injected mice.

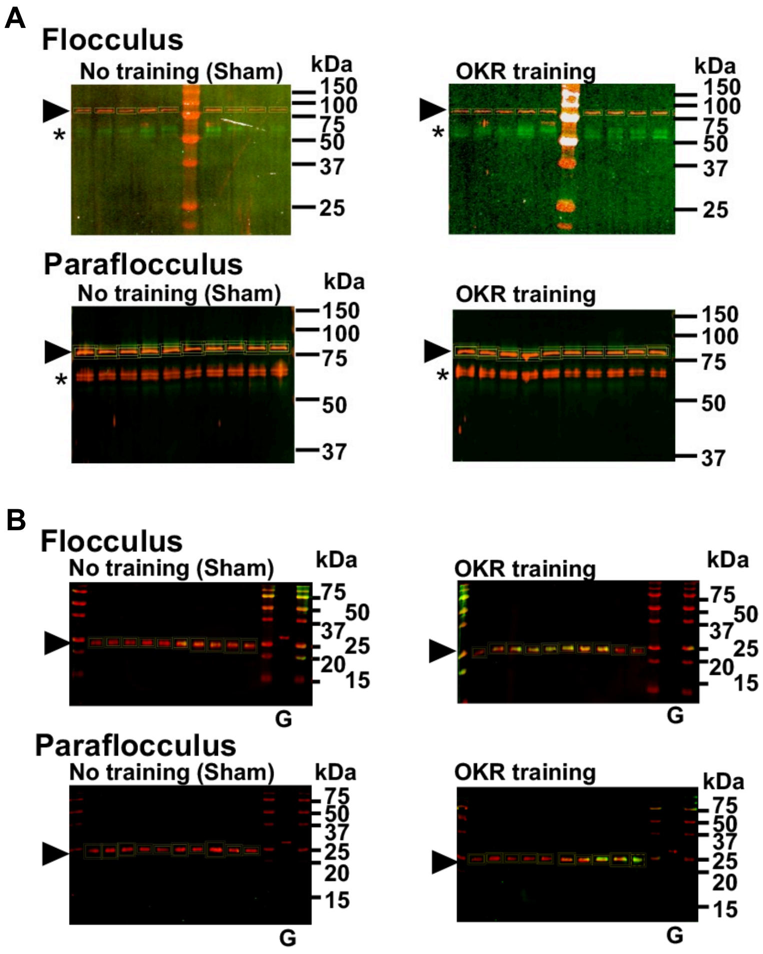

Fig. S4. Activation of PKG–G-substrate pathway by cerebellar learning.

(A) Complete extent of images of Western blots shown in Fig.5A. S-guanylation of PKG1α in flocculi (n=9) and paraflocculi (n=10) induced by OKR training. After the mice were subjected to 4-hr OKR training, flocculi and paraflocculi tissues were harvested and PGK1α was pulled down using AET-cGMP-gel and analysed as described in Methods. For each band in the Western blot, the fluorescence signal for S-guanylated PKG1α (green) was normalized to the signal for PKG1α (red) and plotted in graphs as an arbitrary unit for S-guanylated-PKG/PKG (Fig. 5). One sample (OKR) was removed from further analysis because of the high background observed in the blot.

(B) Complete extent of images of Western blots shown in Fig. 5B. Phosphorylation of G-substrate in flocculi (n=10) and paraflocculi (n=10) induced by optokinetic response (OKR) training. After the mice were subjected to 4-hr OKR training, flocculi and paraflocculi were harvested and processed for the isolation of PKGIα using AET-cGMP gel. The material unbound to the AET-cGMP gel was processed as described in the Methods and subjected to G-substrate phosphorylation analyses. For each band on the Western blot, the fluorescence signal for phospho-G-substrate was normalized using the signal for G-substrate and plotted as an arbitrary unit for phospho-G-substrate/G-substrate (see Fig. 5).

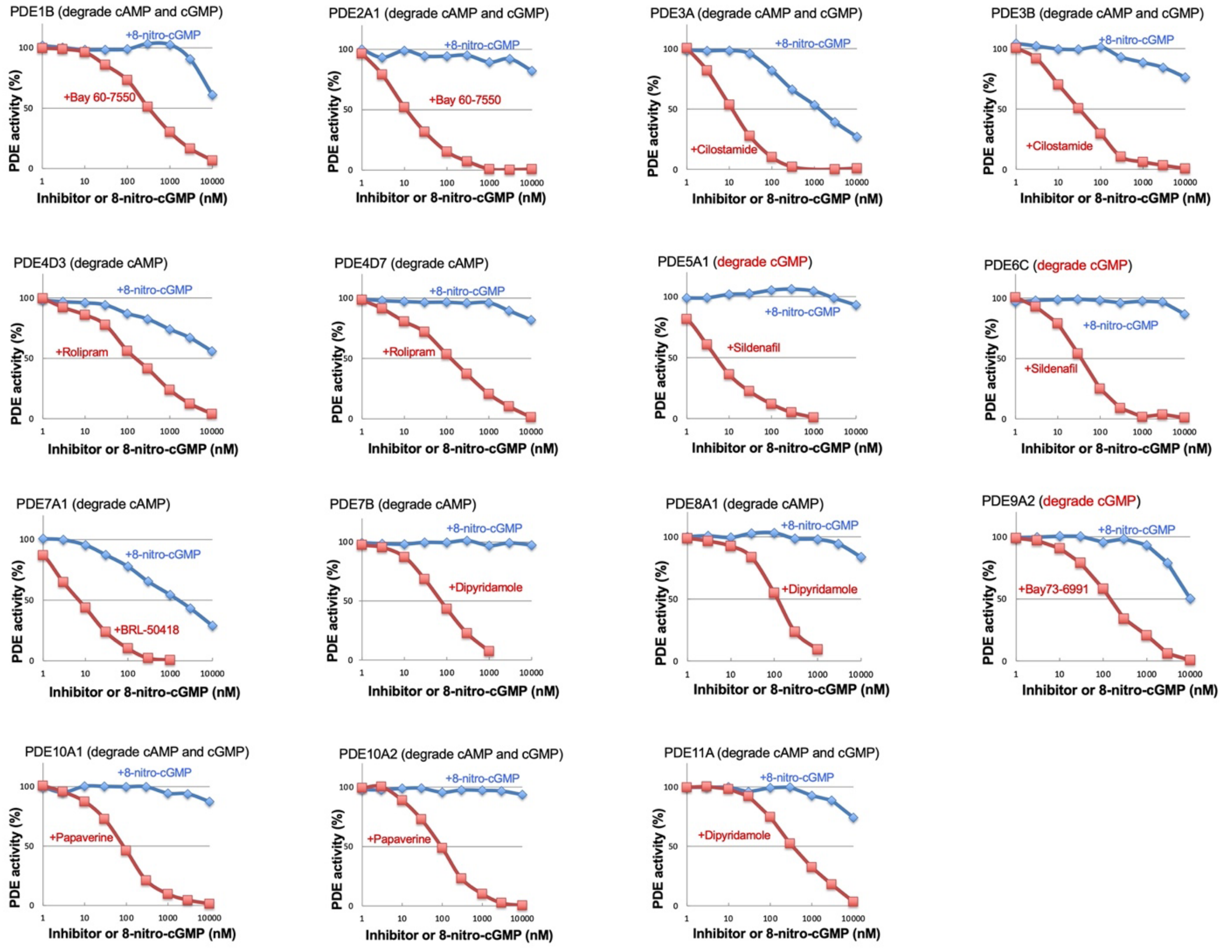

Fig. S5. Phosphodiesterase (PDE) inhibition by 8-NO_2-cGMP.

Inhibition of PDE activity was examined by fluorescence polarization using FAM-labeled cAMP or cGMP as a substrate in the presence of 8-NO_2-cGMP or a specific PDE inhibitor (1-10000 nM). Complete list of PDE examined (amount of PDE, a specific inhibitor used) and the substrate (FAM-cAMP（0.1mM) or FAM-cGMP（0.1mM)) used were as follows: PDE1B (0.5 ng, Bay 60-7550 [Cayman Chemicals, Ann Arbor, MI, USA]), FAM-cAMP; PDE2A1 (0.08 ng, Bay 60-7550 [Cayman Chemicals, Ann Arbor, MI, USA]), FAM-cAMP; PDE3A (0.05 ng, cilostamide [Axxora, San Diego, CA, USA]), FAM-cAMP; PDE3B (0.12 ng, cilostamide [Axxora, San Diego, CA, USA]), FAM-cAMP; PDE4D3 (0.072 ng, rolipram [A.G. Scientific, San Diego, CA, USA]), FAM-cAMP; PDE4D7 (0.045 ng, rolipram [A.G. Scientific, San Diego, CA, USA]), FAM-cAMP; PDE5A1 (0.2 ng, sildenafil [Axxora, San Diego, CA, USA]), FAM-cGMP; PDE6C (22.5 ng, sildenafil [Axxora, San Diego, CA, USA]), FAM-cGMP; PDE7A1 (0.2 ng, BRL-50481[Enzo Life Sciences, Farmingdale, NY, USA]), FAM-cAMP; PDE7B (0.75 ng, dipyridamole [Axxora, San Diego, CA, USA]), FAM-cAMP; PDE8A1 (0.06 ng, dipyridamole [Axxora, San Diego, CA, USA]), FAM-cAMP; PDE9A2(0.0012 ng, Bay 73-6991[Sigma-Aldrich, St. Louis, MO, USA]), FAM-cGMP;

PDE10A1 (0.02 ng, papaverine [Axxora, San Diego, CA, USA]), FAM-cAMP; PDE10A2 (0.008 ng, papaverine [Axxora, San Diego, CA, USA]), FAM-cAMP; PDE11A4 (1.15 ng, dipyridamole [Axxora, San Diego, CA, USA]), FAM-cAMP.

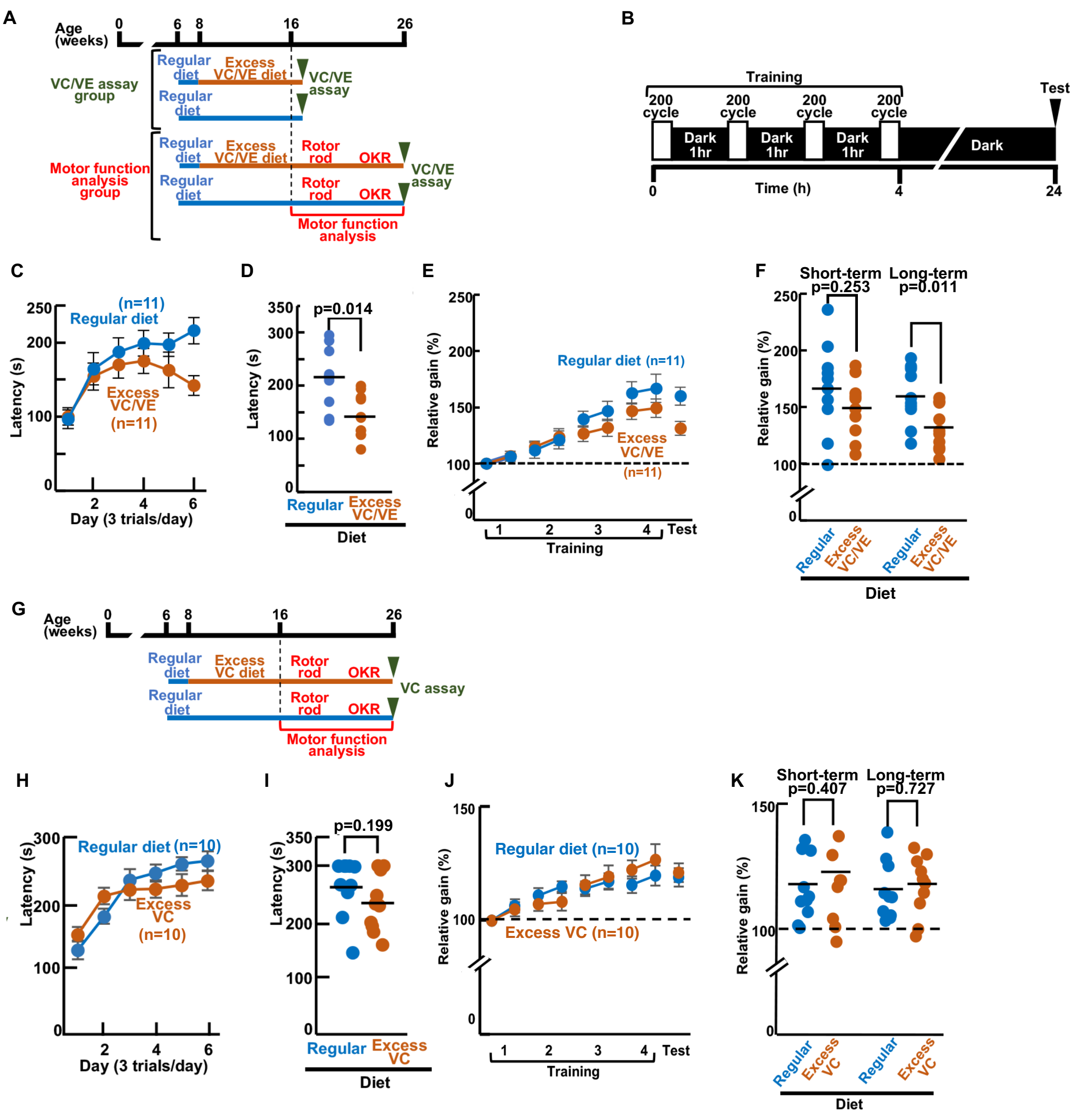

Fig. S6. Inhibition of cerebellar learning by excessive ingestion of vitamins C and E by mice.

(A) Schedule for delivering excessive vitamin C (VC; ascorbate)/vitamin E (VE; α-tocopherol) in the diet of mice and analyses for motor coordination and motor memory. After 8-weeks of the diet administration containing excess VC and VE (VC/VE) or the regular diet, mice were behaviorally assessed using the rotor rod test followed by OKR training and testing. Before (18 weeks, the VC/VE assay group) and the end of experiments (26 weeks, the motor function analysis group), the mice were euthanized, and the amounts of VC and VE in cerebellum were measured (see Table S4). **(B)** Training protocol for OKR. OKR training consisted of four sets of 200 cycles of sustained sinusoidal screen oscillation at 15° and 0.17 Hz with a 1 hr rest in the dark between the sets. Short-term OKR adaptation was examined at the end of the 4-hr training. Mice were kept in the dark in their cages after training. Long-term OKR adaptation was examined 24 hr after the end of the 4-hr training. **(C)** Fall-off latencies in the rotor rod test for mice with or without excessive VC/VE ingestion. Values ± standard error of the mean (SEM). **(D)** Rotor rod performance on the sixth day of the training. **(E)** Comparison of OKR

relative gain measured during 4-hr training (Training 1-4) and that measured after the end of the 4-hr training (Test). Values ± SEM. **(F)** Distribution of relative gains of individual mice at the end of the 4-hr training (short-term) and gains measured after the end of the 4-hr training (long-term). Horizontal bars represent the group mean relative gain. **(G)** Schedule for the administration of excess VC (ascorbic acid), and analyses of motor coordination and motor memory. After 8 weeks of excessive VC administration, the mice were behaviorally assessed using the rotor rod test followed by OKR, as before. Amounts of VC in the cerebellum and plasma were measured as before (see Table S5). **(H)** Fall latencies in the rotor-rod test for mice with or without excessive VC ingestion. Values ± SEM. **(I)** Rotor rod performance on the sixth day of the training. **(J)** Comparison of OKR relative gains measured after 4-hr OKR training (Training 1-4) and that measured 24 hr after the OKR training (Test). Values ± SEM. **(K)** Distribution of relative gains of individual mice at the end of the 4-hr training (short-term) and that measured at 24 hr after the end of the 4-hr training (long-term). Horizontal bars represent group means for relative gain.

Table S1.

Number of subjects used in this study and sample size per experimental condition.

	Diet condition	
	Regular	Excess VC/VE
Vitamin C/E assay with excess VC/VE		
-8-week administration	10	10
-18-week administration	10	10
Behavioral experiments		
Excess VC/VE, excess VC or VE experiments		
-Rotor rod, OKR, then VC/VE assay	20	20
-OKR with flocculus injection experiments	22	
Biochemical experiments		
-PKG S-guanylation, G-substrate phosphorylation evoked	20	
Histochemistry		
-Distribution of 8-nitro-cGMP and S-guanylated proteins	10	
-Induction of immediate early genes by OKR	14	
Electrophysiology & imaging	40	

Table S2.

Standard diet constituents (CRF-1) used in this research.

Nutritional Contents	CRF-1 (g/100g diet)
Water (g)	8.1
Protein (g)	22.3
Fat (g)	5.3
Mineral (g)	6.5
Fiber (g)	3.4
Nitrogen-free extract (g)	54.4
Calorie (kcal)	354.9
Vitamins	
A (IU)	3010
D3 (IU)	580
E (mg)	20.8
K3 (mg)	0.16
B1 (mg)	4.96
B2 (mg)	3.3
C (mg)	16.6
B6 (mg)	1.3
B12 (μg)	15
Inositol (mg)	468.4
Biotin (μg)	34.9
Pantothenate (mg)	4.83
Niacin (mg)	15.66
Choline (g)	0.29
Folate (mg)	0.29

Table S3

The Excess VC/VE diet condition used in this research.

Diet condition	VC (mg /100 g diet)	VE (mg /100 g diet)
Regular	16.6<br>+bottled water (10 μM EDTA)	20.8
Excess VC	16.6<br>+ bottled water (1.5 g/L VC, 10 μM EDTA)	20.8
Excess VE	16.6<br>+bottled water (10 μM EDTA)	54.0
Excess VC/VE	16.6<br>+bottled water (1.5 g/L VC, 10 μM EDTA)	54.0

Table S4.

Contents of VC and VE in the cerebellum of mice fed with diet containing excess VC and VE.

	Duration of VC/VE administration			
	8 weeks		18 weeks	
	Regular diet (μg / g tissue)	Excess VC/VE (μg / g tissue)	Regular diet (μg / g tissue)	Excess VC/VE (μg / g tissue)
Vitamin E	11.2 ± 1.1 (10)	13.0 ± 1.2** (10)	13.4 ± 0.9 (10)	16.4 ± 2.7** (10)
Vitamin C	3.2 ± 0.3 (10)	3.4 ± 0.3 n.s. (10)	3.6 ± 0.2 (10)	3.7 ± 0.3 n.s. (10)

The mice were fed diet as shown in Fig. S6. Regular diet (CRF-1) contains 10 mg/100 g of VC and 20 mg/100 g of VE. The Regular diet group mice were fed regular diet and water. For Excess VC/VE group, were fed the excess amount of both VC (1.5 g/L in water containing 10 μM EDTA) and VE (54 mg/100 g diet) for 8 weeks or 18 weeks. After the 8-week administration for VC/VE assay group or 18-week administration for motor function analysis group, cerebella were collected from the mice and VC and VE were assayed. VC is composed of ascorbic acid and dehydroascorbic acid. VE is α-tocopherol, a major component of VE. Value ± SEM. Numbers in parenthesis indicate the number of mice used. Student's t-test was carried out between the Regular diet group and the Excess VC/VE group. $**p<0.01$ compared with the corresponding regular diet group. n.s., not significant ($p>0.05$).

Table S5.

Amounts of Vitamin C and Vitamin E in the cerebellum and plasma of the mice fed with diet containing excess either VC or VE.

	Diet condition		
Vitamin/Tissue	Regular	Excess VC	Excess VE
Vitamin E amount			
Cerebellum (μg/ g tissue)	12.7 ± 0.4 (10)	n.d.	13.8 ± 0.4 (10)*
Plasma (μM)	6.8 ± 0.4 (10)	n.d.	11.1 ± 1.0 (10)***
Vitamin C amount			
Cerebellum (μg/g tissue)	4.0 ± 0.1 (10)	4.3 ± 0.1 (10)***	n.d.
Plasma (μM)	86.3 ± 2.9 (10)	93.6 ± 4.0 (10) n.s.	n.d.

The Regular diet group mice were fed regular diet (CRF-1) and water containing 10μM EDTA. For Excess VC or VE group, were fed the excess amount of either VC (1.5 g/L in water containing 10 μM EDTA) or VE (54 mg/100 g diet) for 18 weeks (see Fig. 6A and Fig. S6A, G). After the motor function analysis, cerebella and plasma were collected from the mice and VC and VE were assayed at 26 weeks. VC is composed of ascorbic acid and dehydroascorbic acid. VE is assayed as α-tocopherol, a major component of VE. Value ± SEM. Numbers in parenthesis indicate the number of mice used for the assay. Student's t-test was carried out between the Regular diet group and the Excess VC or VE group. n.s., not significant. *p<0.05, **p<0.01, ***p<0.001 compared with the corresponding regular diet group. n.d., not determined. n.s., not significant (p>0.05).

Table S6.

Body weight and diet consumption of the mice fed with Excess VC diet and Regular diet.

Diet	Body weight (g)		Daily diet consumption per cage (g)	
	Initial	8-week administration	Initial	8-week administration
	n.s.	n.s.	#	#
Regular diet (10)	22.7 ± 0.2	28.7 ± 0.5	13.8 ± 0.2	14.3 ± 0.1
Excess VC (10)	22.6 ± 0.3	28.8 ± 0.4	14.7 ± 0.7	15.1 ± 0.9

The mice in the Regular diet group were provided with a standard diet (CRF-1; see Table S2) and water containing 10 μM EDTA. In contrast, the mice in the Excess VC group were fed the same CRF-1 diet along with an additional amount of VC (1.5 g/L) in water containing 10 μM EDTA (see Table S3 for detail). The feedings of these diet were carried out for a duration of 8 weeks, starting when the mice were 8 weeks old (referred to as the "Initial" time point in this table; please also refer to Fig. 6A). Body weight and diet consumption were measured at the beginning and after the 8-week administration. The values reported are presented as mean ± SEM, and the number of mice used for each measurement is indicated in parentheses. To compare the body weight between the Regular diet group and the Excess VC group, Student's t-test was performed for the comparison of body weight between the Regular diet group and the Excess VC group at the beginning and after 8-week administration. #The diet consumption was not subjected to statistical analyses due to the fact that the reported values represent the averaged values of daily consumption per cages (5 mice/cage; n=2 cages). In the statistical analysis, "n.s." denotes that the observed differences were not significant between Excess VC diet group and Regular diet group (p>0.05).
